# The Diversity, Metabolomics Profiling, and the Pharmacological Potential of Actinomycetes Isolated from the Estremadura Spur Pockmarks (Portugal)

**DOI:** 10.3390/md20010021

**Published:** 2021-12-23

**Authors:** António Pinto-Almeida, Anelize Bauermeister, Luca Luppino, Inês R. Grilo, Juliana Oliveira, Joana R. Sousa, Daniel Petras, Clara F. Rodrigues, Alejandra Prieto-Davó, Deniz Tasdemir, Rita G. Sobral, Susana P. Gaudêncio

**Affiliations:** 1 Associate Laboratory i4HB—Institute for Health and Bioeconomy, NOVA School of Science and Technology, NOVA University Lisbon, 2819-516 Caparica, Portugal; pintoalmeida84@gmail.com (A.P.-A.); luppinoluca00@gmail.com (L.L.); inesgrilo@fct.unl.pt (I.R.G.); jpc.oliveira@campus.fct.unl.pt (J.O.); jrl.sousa@campus.fct.unl.pt (J.R.S.); rgs@fct.unl.pt (R.G.S.); 2 UCIBIO—Applied Molecular Biosciences Unit, NOVA School of Science and Technology, NOVA University of Lisbon, 2819-516 Caparica, Portugal; 3 Instituto de Engenharias e Ciências do Mar, Universidade Técnica do Atlântico, 163 Ribeira de Julião, 163 Mindelo, Cape Verde; 4 Skaggs School of Pharmacy & Pharmaceutical Science, University of California San Diego, La Jolla, CA 92093-075, USA; abauermeister@health.ucsd.edu; 5 Dipartimento di Scienze Della Vita, Università Degli Studi di Modena e Reggio Emilia, 41125 Modena, Italy; 6 CMFI Cluster of Excellence, Interfaculty Institute of Microbiology and Medicine, University of Tuebingen, Auf der Morgenstelle 24, 72076 Tuebingen, Germany; daniel.petras@uni-tuebingen.de; 7 CESAM—Centre for Environmental and Marine Studies, Department of Biology, University of Aveiro, 3810-193 Aveiro, Portugal; clara.rodrigues@ua.pt; 8 Unidad de Química-Sisal, Facultad de Química, Universidad Nacional Autónoma de México, Sisal 97356, Mexico; apdavo@unam.mx; 9 GEOMAR Centre for Marine Biotechnology, Research Unit Marine Natural Products Chemistry, GEOMAR Helmholtz Centre for Ocean Research Kiel, 24106 Kiel, Germany; dtasdemir@geomar.de; 10 Faculty of Mathematics and Natural Sciences, Kiel University, Christian-Albrechts-Platz 4, 24118 Kiel, Germany

**Keywords:** marine-derived actinomycetes, actinobacteria, Estremadura Spur pockmarks, molecular networking, metabolomics, Qemistree, antimicrobial activity, marine natural products, blue biotechnology

## Abstract

The Estremadura Spur pockmarks are a unique and unexplored ecosystem located in the North Atlantic, off the coast of Portugal. A total of 85 marine-derived actinomycetes were isolated and cultured from sediments collected from this ecosystem at a depth of 200 to 350 m. Nine genera, *Streptomyces*, *Micromonospora*, *Saccharopolyspora*, *Actinomadura*, *Actinopolymorpha*, *Nocardiopsis*, *Saccharomonospora*, *Stackebrandtia*, and *Verrucosispora* were identified by 16S rRNA gene sequencing analyses, from which the first two were the most predominant. Non-targeted LC-MS/MS, in combination with molecular networking, revealed high metabolite diversity, including several known metabolites, such as surugamide, antimycin, etamycin, physostigmine, desferrioxamine, ikarugamycin, piericidine, and rakicidin derivatives, as well as numerous unidentified metabolites. Taxonomy was the strongest parameter influencing the metabolite production, highlighting the different biosynthetic potentials of phylogenetically related actinomycetes; the majority of the chemical classes can be used as chemotaxonomic markers, as the metabolite distribution was mostly genera-specific. The EtOAc extracts of the actinomycete isolates demonstrated antimicrobial and antioxidant activity. Altogether, this study demonstrates that the Estremadura Spur is a source of actinomycetes with potential applications for biotechnology. It highlights the importance of investigating actinomycetes from unique ecosystems, such as pockmarks, as the metabolite production reflects their adaptation to this habitat.

## 1. Introduction

Pockmarks are seabed culminations of fluid (liquid or gas) migrations through the sedimentary column and their escape to the seawater, which appear as cone-shaped circular or elliptical depressions [1]. Some are active, seeping oil or methane, while others are dormant or inactive. Active pockmarks are well-studied since they harbor specialized microbial communities capable of metabolizing compounds such as methane and other hydrocarbons, and such communities are not found in inactive pockmarks [2]. Inactive pockmarks can present microbial communities that are totally different from the surrounding environment and are poorly studied, highlighting the importance of exploring these ecosystems [2]. The Estremadura Spur pockmark field, off the coast of continental Portugal, is the first documented evidence of fluid seepage in the Lusitanian Basin, a Mesozoic rifted basin containing promising hydrocarbon occurrences [3]. This field of pockmarks exhibits individual pockmarks up to 120 m in diameter and 10 m in depth and is located at a depth of 200 to 350 m. These pockmarks are considered inactive, as they were covered by recent sediments. The stacking of various pockmarks suggests a cyclical fluid flow of activity, possibly resulting from the eustatic sea level variations and changes in hydrostatic pressure [3].

Actinomycetes are a chemically prolific source of bioactive secondary metabolites [4] and are of commercial importance, such as enzymes of industrial interest [5] and lead structures for the development of new drugs [6]. They produce the majority of the existing naturally occurring antibiotics (ca. 75%) [7,8], mostly obtained from soil *Streptomyces* [9]. The genetic and metabolic diversity of marine actinomycetes reflects their adaptation throughout evolution to their particular marine conditions [10,11,12,13,14]. Salinosporamide A (Marizomib^TM^), currently in phase III clinical trials for the treatment of glioblastoma, is the metabolite that better highlights the importance of marine-derived actinomycetes [15,16,17]. The bottom of the ocean is an important source for novel actinomycetes and the associated metabolites [10,11,12,13,14]. Nevertheless, the difficulty in accessing challenging marine environments has hindered the exploration of their biodiversity and their distribution along the seafloor is still largely unknown. The description of indigenous marine actinomycetes in the ocean remains underexplored.

This study aimed to explore the biological and chemical diversity of cultivable actinomycetes from the Estremadura Spur pockmarks, and to evaluate their ability to produce metabolites with antimicrobial (antibacterial, antiyeast, and antifungal), anticancer, and antioxidant activities. This is the first study reporting the targeted culture-dependent identification of actinomycetes from the Estremadura Spur pockmarks, their metabolomics profile, and their biotechnological potential.

## 2. Results and Discussion

### 2.1. Cultivable Actinomycetes’ Phylogeny and Diversity

**Figure 1:**
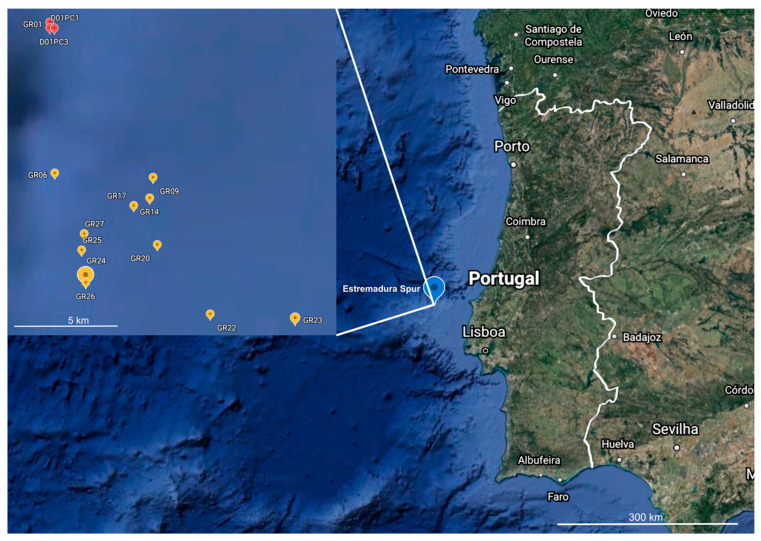
Geographical location of the Estremadura Spur, continental Portugal coast, with the locations of the marine sediment collection stations zoomed in. The collection points marked in red correspond to small mounds and those in yellow correspond to pockmarks. This map was retrieved from Google Earth (https://earth.google.com/web/ accessed on 25 September 2021).

A total of 85 actinomycete isolates were cultivated from the Estremadura Spur sediments (Figure 1) and their 16S rRNA sequences were compared to the NCBI and EzTaxon databases. No chimeric sequences were identified.

**Figure 2:**
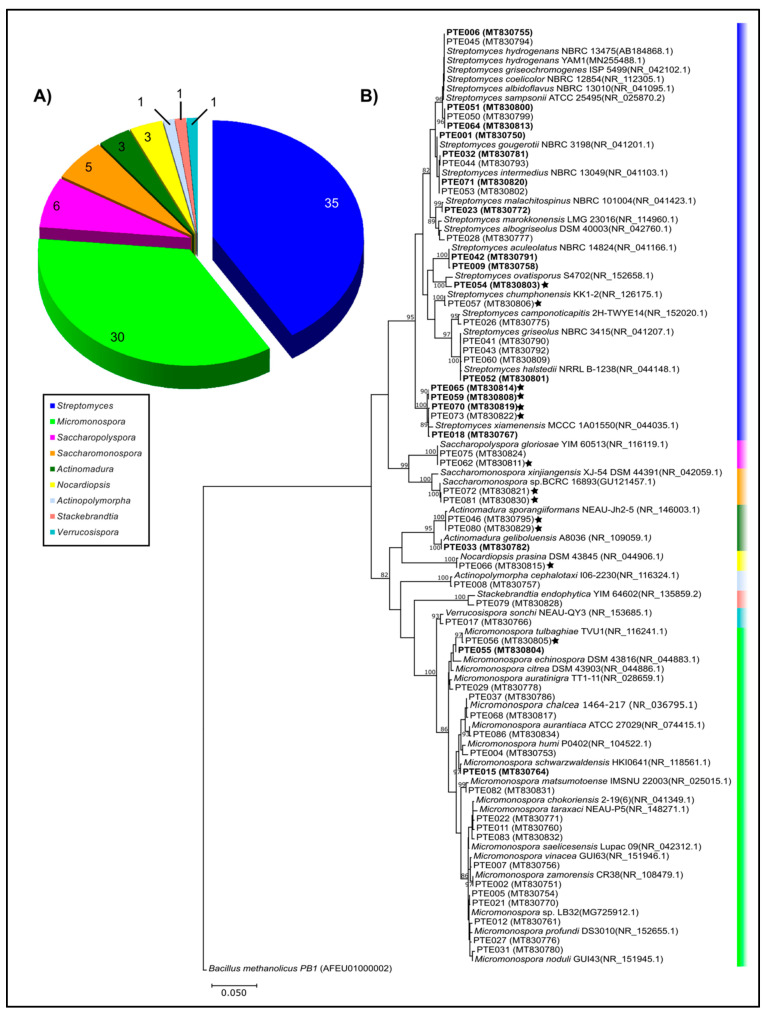
(**A**) Abundance of actinomycetes genera isolated from Estremadura Spur continental Portugal coastal marine sediments, with indication of the absolute numbers. (**B**) Maximum likelihood phylogenetic tree of the alignment of the 16S rRNA gene sequences from the 53 representative actinomycetes OTUs from Estremadura Spur, continental Portugal, and the representative sequences of all species retrieved from the GenBank (Table S2). The tree was created using 1000 bootstraps. Nodes above 80% bootstrap values are shown. GenBank accession numbers are indicated after the sequence name. All PTE numbers refer to internal reference collection codes. Stars symbol represent the strains with seawater requirement for growth. Bioactive PTEs strains are shown in bold. *Bacillus methanolicus* was used as outgroup.

For the taxonomic classification at the species level of some isolates, further phylogenetic analyses were used for clarification (see the “Materials and Methods” in Section 3 and Figure S1 in Supplementary Materials). Overall, nine different genera were discovered: *Streptomyces, Micromonospora, Verrucosispora, Nocardiopsis*, *Actinomadura*, and the rare genera *Saccharomonospora,* as well as *Saccharopolyspora, Stackebrandtia,* and *Actinopolymorpha*. The genera *Streptomyces* and *Micromonospora* are by far the most abundant ones (Figure 2A). The high abundance of these two genera agrees with previous studies [6,18,19,20,21,22], including work from our group that was performed with actinomycetes collected off the Madeira Archipelago, Portugal [23], suggesting that these genera are very abundant in all oceans, or the media used in the reported studies selects for them. In total, 21.2% (*n* = 85) of the Estremadura Spur isolates required seawater for growth, suggesting that they are obligate marine actinomycetes (Figure 2B and Figure S1, highlighted with black stars, and Table S3). Although marine obligate *Salinispora* strains have been recovered from shallow water samples from the Madeira Archipelago, Portugal [23,24], this genus was not recovered from the Estremadura Spur sediments, which also supports the hypothesis that these pockmarks present a differentiated microbial community.

Different media were used to isolate the actinomycetes. The A1 medium allowed the isolation of only six strains belonging to *Streptomyces*, *Micromonospora,* and *Verrucosispora*, reducing concentrations to one half (½ A1 medium), which allowed the growth of a higher number of strains (24 strains) from *Streptomyces*, *Micromonospora,* and *Saccharopolyspora*. The SWA medium allowed the highest number of actinomycete isolates, 56 strains, from all the obtained genera, except *Verrucosispora*. The isolation of actinomycetes was clearly favored by the SWA medium.

Fifty-three operational taxonomic units (OTUs) were defined (at 97% sequence identity with their nearest BLASTn neighbor) under the nine different genera of cultivated actinomycetes (Table 1, Figure 2A). From these, 24 OTUs (35 isolates) belonged to *Streptomyces* and 19 OTUs (30 isolates) to *Micromonospora*.

The phylogenetic tree revealed that the higher recovery of these two genera resulted in higher microbial diversity within them (Figure 2B). The remaining genera were represented each by a single branch in the 16S rRNA molecular phylogenetic tree, suggesting that these actinomycete genera are either less adaptable to the used cultivation conditions, or that their microbial diversity in the Estremadura Spur sediments is low. All strains had more than a 98% sequence identity with their nearest BLASTn neighbor. However, six out of the nine genera recovered by cultivation included strains with seawater requirements for growth (Figure 2B), suggesting they could well represent different species from those neighbors. This finding poses new questions about the evolution of marine actinomycete strains in the Atlantic Ocean, as this is the third study in which our group found a high abundance of seawater requiring strains from these waters [23,24,25]. Furthermore, seawater-requiring strains are found in more than 50% of the recovered genera, while work from other oceanic locations describes this trait as one that has mostly been reported for *Streptomyces* and *Salinispora* [14,21,26,27,28,29]. Amongst these “marine obligate actinomycetes”, a clade formed by five *Streptomyces* strains, closely related to *S. xiamenensis* (NR044035.1), highlights the importance of studying microbial diversity from ocean sediments (Figure 2B). In this clade, strains with identical 16S rRNA sequences not only show different growth requirements, but also differences in the production of bioactive metabolites in their extracts (see below).

**Figure 3:**
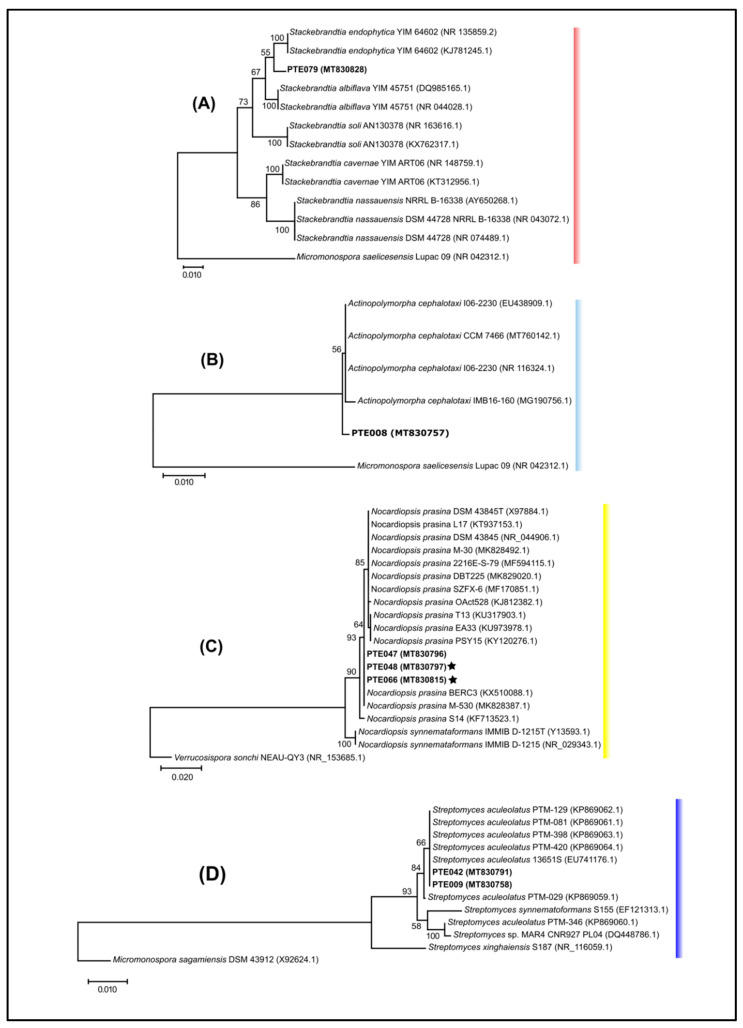
Maximum likelihood phylogenetic tree reconstruction of the alignments of the 16S rRNA gene for four different actinomycetes species from the Estremadura Spur, continental Portugal. (**A**) *Stackebrandtia endophytica*, (**B**) *Actinopolymorpha cephalotaxi*, (**C**) *Nocardiopsis prasina*, and (**D**) *Streptomyces aculeolatus*. Each alignment was comprised of sequences of the isolates from the respective species found in this study and the sequences of the same species available in GenBank. Trees were created using 1000 bootstraps. Nodes above 50% bootstrap values are shown. GenBank accession numbers are indicated after the sequence name. All PTE numbers refer to internal reference collection codes. Stars symbol represent the strains with seawater requirement for growth.

To further explore the evolutionary relationships between our strains and the previously reported ones, we inspected strains related to *Nocardiopsis prasina, Actinopolymorpha cephalotaxi, Stackebrandtia endophytica,* and *Streptomyces aculeolatus* (Figure 3), given the relevance of some *N. prasina* enzymes (chitinases and serine protease) in industrial processes [30,31]; the rarity of *A. cephalotaxi* [32] and *S. endophytica* [33,34], which represent poorly studied species; and the bioactive meroterpenoids produced by *S. aculeolatus* which are compound families with therapeutic and industrial potential [23,35,36] and were previously isolated from the Madeira Archipelago [25,36].

Our results revealed that the isolates PTE-008 (MT830757) and PTE-079 (MT830828) from the Estremadura Spur had a 99% sequence identity with *A. cephalotaxi* and *S. endophytica*, respectively. However, the phylogenetic analysis showed that although these isolates cluster with their nearest neighbors, they clade separately into a different branch (Figure 3A,B), suggesting that they could be novel strains. On the other hand, the Estremadura Spur isolates, identified as *N. prasina* (PTE-047 MT830796, PTE-048 MT830797 and PTE-066 MT830815) and *S. aculeolatus* (PTE-009 MT830758 and PTE042 MT830791), had 99% sequence identity with their BLASTn best hits; the phylogenetic analysis revealed clustering within the same branches of the previously described strains, BERC3 (KX510088.1) and M-530 (MK828387.1) for *N. prasina* (Figure 3C), and with the Madeira Archipelago isolates PTM-081 (KP869061.1), PTM-129 (KP869062.1), PTM-398 (KP869063.1) and PTM-420 (KP869064.1) for *S. aculeolatus* (Figure 3D), suggesting they are the same species.

A phylogenetic analysis of the BLASTn nearest neighbors and the isolates from *Streptomyces* and *Micromonospora* genera revealed a close phylogenetic relationship of different isolates and BLASTn neighbors named after different species in the GenBank database (Figure S2), a commonly encountered sequence divergence error from these analyses [37,38,39,40,41,42]. Further phylogenetic analyses with these Estremadura Spur isolates (Figures S3 and S4) confirmed the observed ambiguity of 16S rRNA sequence phylogeny; however, the PTE strains were referred to their nearest BLASTn neighbor for further analyses to simplify identification within GenBank strains.

Rarefaction curves for the diversity estimation, together with the richness estimator Chao 1 [43] (Figure S5), suggest that improving cultivation methods and increasing sampling efforts may improve the recovery of actinomycete OTUs in these sediments. When compared to their nearest neighbors from the GenBank database, PTE-074, PTE-078, and PTE-085 shared less than a 99% similarity in their 16S rRNA sequence identities. However, the metabolomics analyses of these strains showed that the metabolites produced by each of them are either novel or have not been deposited in the GNPS database (see Section 2.2, Section 2.3, Section 2.4 and Section 2.5). Therefore, these strains could be novel OTUs represented by strains which produce unique secondary metabolites that allow them to inhabit specific niches in this environment, and eventually leads them to become novel actinomycete species. This trait has been observed in different *Salinispora* species, which can share over 99% of their 16S rRNA gene identity and still produce distinct compounds that allow them to grow in a wide diversity of environments, turning them into different species [44,45]. These results suggest that the uniqueness of the pockmark environment found in the Estremadura Spur influences the diversity of actinomycetes present in it, and it can thus be a source of novel actinomycetes with a high potential for new chemical compounds.

### 2.2. The Metabolomics Profile of Actinomycete Extracts

**Figure 4:**
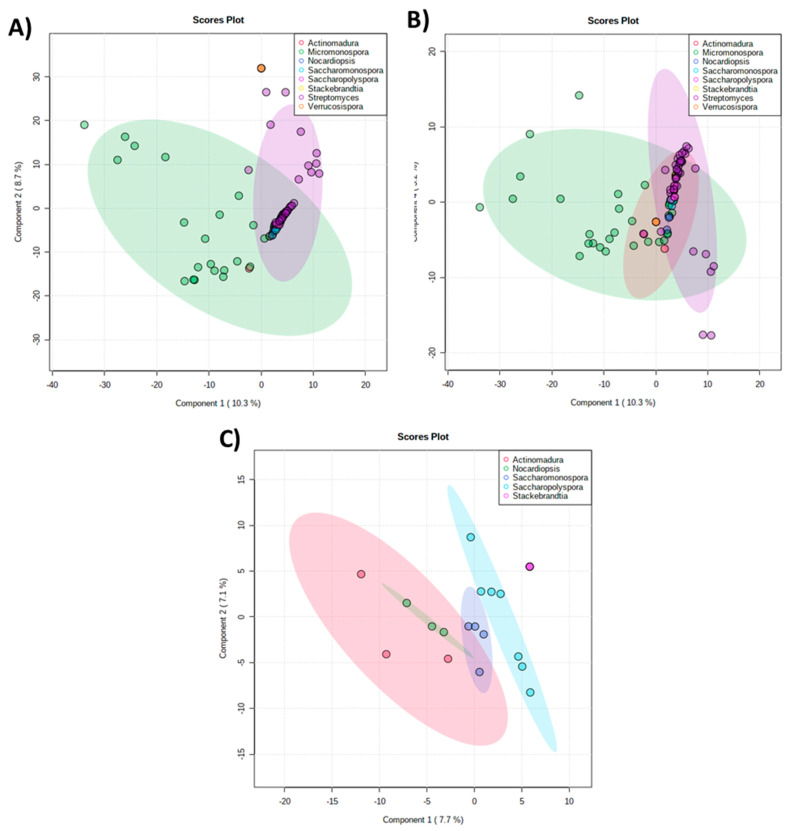
Principal component analysis (PCA). (**A**) Estremadura Spur actinomycetes crude extracts, indicating two predominant clusters (groups *Micromonospora* and *Streptomyces*) formed by chemical similarity. (**B**) Clustering without *Micromonospora* and *Streptomyces* outliners, indicating three main clusters (groups *Micromonospora*, *Streptomyces*, and *Actinomadura*). (**C**) Clustering without *Micromonospora* and *Streptomyces* data, revealing the clusters for *Actinomadura*, *Nocardiopsis*, *Saccharomonospora*, *Saccharopolyspora*, and *Stackebrandtia*.

The metabolic profiling of the actinomycete crude extracts was investigated by an unsupervised principal component analysis (PCA) to evaluate the similarities and differences between the extracts, accessing clustering and trends and identifying outliers. The results obtained from the PCA (Figure 4A) displayed two distinct prevalent groups corresponding to extracts produced by the *Micromonospora* and *Streptomyces* genera, highlighted in green and purple, respectively, which overlapped with all the other genera (Figure 4A,B). This clustering is in accordance with the phylogenetic analysis in which these genera are predominant (Figure 2). By removing these predominant groups (*Micromonospora* and *Streptomyces*) from the PCA, the overlapping region was cleared (Figure 4C), and four distinct groups were revealed by the extracts of *Actinomadura*, *Nocardiopsis*, *Saccharomonospora*, and *Saccharopolyspora*, presented in pink, green, blue, and light blue, respectively. The metabolites produced by *Stackebrandtia* were presented separately from the previous four groups. Interestingly, this clustering highlighted that the strains from these five genera produced distinct chemistry.

**Figure 5:**
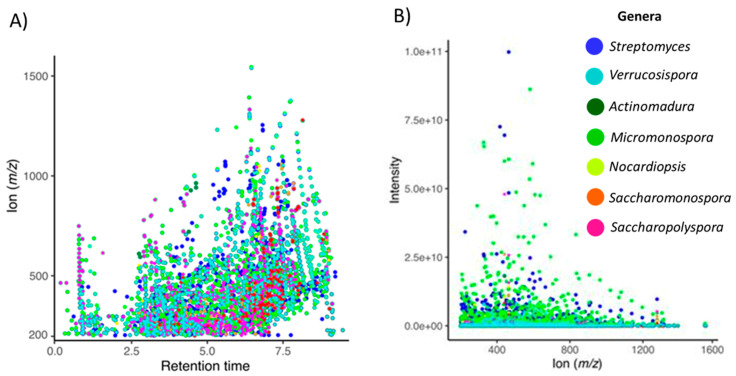
Metabolomics profile of actinomycete crude extracts. (**A**) Scatter plot showing the distribution of the detected ion as *m/z* according to the elution from the chromatographic system. (**B**) Scatter plot showing the ions *m/z* with higher abundances. Colors represent the genera of the strains that produced the extracts according to the legend.

The data shows taxonomy as the strongest parameter that influences the metabolite production, not the habitat or the seawater requirements. Figure 5A displays a trend in which the higher the *m*/*z*, the lower the polarity. Figure 5B shows that the *m/z* in the range of approximately 200–700 Da are the most abundant in the actinomycetes extracts.

### 2.3. MS/MS-Based Molecular Networking

**Figure 6:**
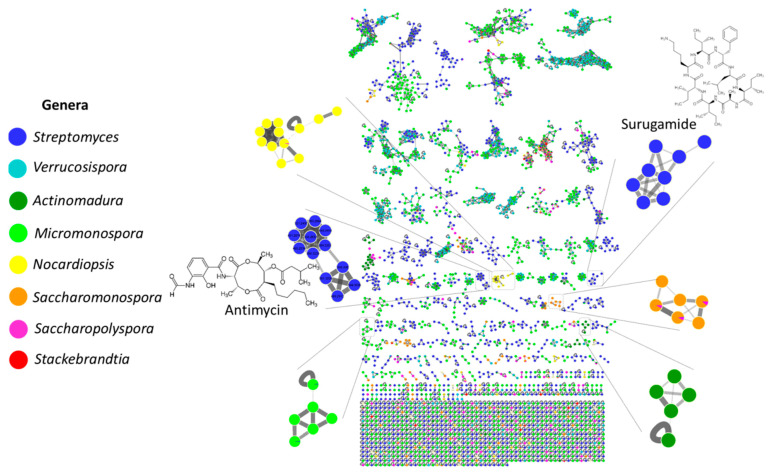
Molecular networking for the Estremadura Spur strains using MS/MS data with a positive ionization mode (ESI+). Nodes represent parent ions, and edge strength indicates the chemical similarity between the MS/MS spectra. Node colors represent the extracts according to the legend. The node size indicates the number of MS/MS spectra. Only clusters containing at least two nodes are shown.

To improve the visualization of the LC-MS/MS data, a molecular network was created in the GNPS platform for the extracts produced by the Estremadura Spur strains (Figure 6) [46,47]. It detected 9025 nodes (ions), of which 392 (approximately 4%) were annotated (a spectral match with known compounds), enabling dereplication [47]. PTE-008, the only strain recovered from the *Actinopolymorpha* genus, did not grow in the used culture conditions, which is why it was left out of the metabolomics profiling analysis and further studies.

Chemical classes that have been previously described [48,49,50,51,52,53], such as peptides (surugamides, etamycins, and rakicidins), alkaloids (physostigmines), polyketides (piericidins), hybrids from the polyketide synthase (PKS)/non-ribosomal peptide synthase (NRPS) biosynthetic pathway (antimycins), hydroxamate-type siderophores (desferrioxamines), and macrolactams (ikarugamycins) were annotated by the GNPS spectral library (level 2, according to MSI) [50]. Surugamides are cyclic octapeptides with the ability to inhibit cathepsin B, a cysteine peptidase overexpressed in several pathological events such as inflammation and cancer; thus, it has good prospects for cancer treatment [51,54,55].

Antimycins are nine-membered bis-lactones exhibiting antibiotic activity that induce apoptosis and inhibit the mitochondrial electron transport chain from cytochrome b to cytochrome C1 [56,57,58,59]. Etamycins are cyclic peptide antibiotics first isolated from *Streptomyces* sp. [48,60,61]. Physostigmines are tricyclic skeleton alkaloids originally isolated from plants [62] that exhibit parasympthomimetic activities and acetylcholinesterase inhibition. Alkaloids belonging to the physostigmine class were first isolated from the marine environment from the bryozoan *Flustra foliaceae* [63]. Desferrioxamides are cyclic trihydroxamate catecholate-type siderophores that have a higher affinity for the chelation of Fe and is being used to treat thalassemia major patients by chelating the overloaded iron [64,65]. Ikarugamycins are polycyclic tetramate macrolactams with diverse biological activities, such as antimicrobial and anti-protozoal activities [66,67]. Piericidines are a well-known class of compounds that have a 4-pyridinol core linked with a methylated polyketide side chain with insecticidal, antimicrobial, and antitumor activities [68]. Rakicidins are macrocyclic lipodepsipeptides that exhibit antibacterial and anticancer activities [69,70]. Interestingly, except for desferrioxamines, the annotated chemical families are very distinct from the actinomycetes isolated from the Madeira Archipelago, Portugal, studied by our group. We annotated nucleoside derivatives, diketopiperazines, phenazines, peniprequinolones, and desferrioxamines [23] which, as mentioned above, also showed *Streptomyces* and *Micromonospora* as the main prevalent genera, highlighting that the Estremadura Spur pockmarks are a very interesting and unique source of actinomycetes, and consequently, metabolites. Conversely, the metabolites annotated from the actinomycetes from the Estremadura Spur exhibited similar chemistry to those obtained from Rocas Atoll, Brazil (a unique atoll in the South Atlantic) to some extent, in which surugamides and antimycins were detected, suggesting a wide distribution of their *Streptomyces* producers in the Atlantic Ocean [55].

The molecular network allowed the visualization of several molecular families that are produced exclusively by specific taxonomic groups (genus-specific), such as surugamides, antimycins, and etamycins produced by *Streptomyces* strains, as well as physostigmines, desferrioxamines, ikarugamycins, piericidines, rakicidins, and many others that have not been identified. The MS/MS results revealed that the number of exclusive metabolites produced by the strains obtained from the pockmarks is higher than those obtained from small mound strains (Figure S6A). However, these numbers might not be relevant, as the number of small mound stations and isolated strains is smaller than those from the pockmarks (Table 2 and Table S1).

The produced metabolites are more likely strain-specific than marine-obligate (Figure 5 and Figure S6A,B). Nevertheless, several unknown molecular families have been detected exclusively in seawater-requiring strains (Figure S7), as was the case for *Micromonospora saelicesensis* PTE-038, *Streptomyces ovatisporus* PTE-054, *Streptomyces chumphonensis* PTE-057, *Streptomyces xiamenensis* PTE-059, *Streptomyces chumphonensis* PTE-061, *Streptomyces xiamenensis* PTE-065, *Streptomyces xiamenensis* PTE-070, *Saccharomonospora xinjiangensis* PTE-072, *Saccharomonospora piscinae* PTE-081, and *Saccharomonospora xinjiangensis* PTE-085.

### 2.4. Surugamide, Antimycin, Etamycin, Physostigmine, Desferrioxamine, Ikarugamycin, Piericidine, and Rakicidin Families

**Figure 7:**
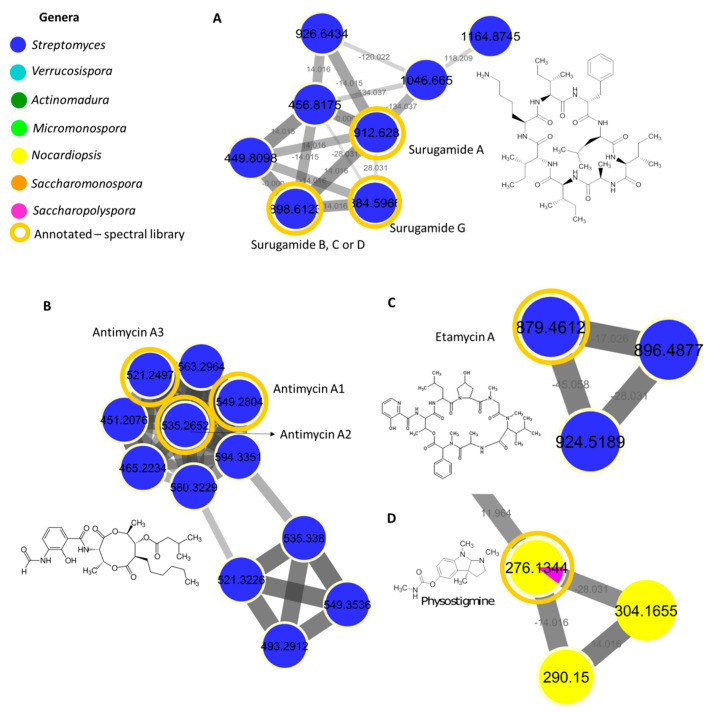
(**A**) Cluster from surugamides produced by *Streptomyces* strains. Nodes represent parent ions, and edge strength indicates the chemical similarity between the MS/MS spectra. Node colors represent the extracts according to the legend. The node size indicates the number of MS/MS spectra. Only clusters containing at least two nodes are shown. (**B**) Cluster from antimycin derivatives produced by *Streptomyces* strains. (**C**) Cluster from etamycin derivatives produced by *Streptomyces* strains. (**D**) Cluster from physostigmine derivatives produced by *Nocardiopsis* strains. The structures are representatives of each molecular family. MS does not detect stereochemistry, except a few particular cases.

Our results revealed unknown surugamide, antimycin, etamycin, physostigmine, desferrioxamine, ikarugamycin, piericidine, and rakicidin derivatives, suggesting the Estremadura Spur to be a promising source of new compounds for future investigation with prospects that can be applied for medical and biotechnological uses. Surugamide derivatives were found in seven *Streptomyces* strains: PTE-035, PTE-036, PTE-040, PTE-045, PTE-050, PTE-054, and PTE-064. To date, there are only five surugamides described in the literature [51,71]. The surugamide family contains several unknown derivatives (Figure 7A), thus suggesting that these actinomycetes are a promising source of new compounds to be further investigated. Antimycins were detected in strains PTE-001, PTE-035, PTE-036, PTE-040, PTE-045, PTE-049, PTE-050, and PTE-064 from *Streptomyces* genera (Figure 7B) and several metabolites detected in these strains are unknown. Etamycin derivatives were detected in the *Streptomyces* strain PTE-054 (Figure 7C). Comparing the etamycin node data with the literature, we suggest that some of these derivatives might belong to the fijimycin class. Fijimycins are etamycin-class depsipeptides that exhibit antibacterial activity [72]. To date, only three fijimycins have been reported [72].

Although originally isolated from the *Streptomyces* species, in our work, physostigmine derivatives were detected in strains PTE-019, PTE-021, PTE-030, PTE-047, PTE-048, PTE-049, PTE-054, and PTE-066 belonging to *Nocardiopsis*, *Micromonospora* and *Saccharopolyspora* genera (Figure 7D). Desferrioxamine derivatives were detected in strains PTE-007, PTE-030, PTE-031, PTE-055, PTE-061, PTE-075, and PTE-076 from *Micromonospora*, *Streptomyces* and *Saccharopolyspora* genera. Rakicidin derivatives were detected in strains PTE-024, PTE-065, PTE-075, and PTE-076 also belonging to *Micromonospora*, *Streptomyces* and *Saccharopolyspora* genera. Ikarugamycin derivatives were present in numerous strains, such as PTE-018, PTE-019, PTE-033, PTE-034, PTE-038, PTE-046, PTE-053, PTE-054, PTE-059, PTE-060, PTE-061, PTE-063, PTE-064, PTE-065, PTE-066, PTE-067, PTE-068, PTE-071, PTE-073, PTE-074, and PTE-085 from *Streptomyces*, *Saccharopolyspora*, *Micromonospora*, *Actinomadura*, *Nocardiopsis*, and *Saccharomonospora* genera. Finally, piericidine derivatives were also present in several strains, namely, PTE-024, PTE-027, PTE-034, PTE-035, PTE-036, PTE-041, PTE-054, PTE-056, PTE-057, PTE-058, PTE-061, and PTE-085 from *Streptomyces*, *Micromonospora*, and *Saccharomonospora* genera.

Besides the molecular families specifically shown in the *Streptomyces* and *Nocardiopsis* strains (Figure 7), the Estremadura Spur actinomycetes revealed numerous other molecular families that were detected in a single genus (i.e., genera-specific) which have not been annotated by the GNPS, as was the case of *Streptomyces*, *Micromonospora*, *Actinomadura*, *Saccharopolyspora*, and *Saccharomonospora*. Overall, this clustering is in accordance with the genera grouping observed in the PCA and denotes the high chemical potential of these strains for the discovery of novel secondary metabolites.

### 2.5. Qemistree Data Analysis

**Figure 8:**
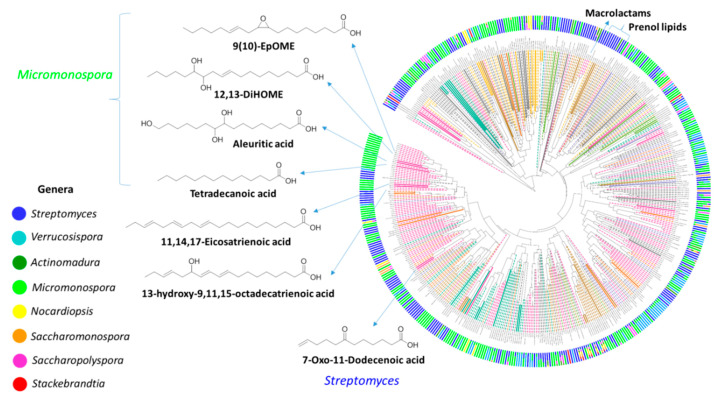
Estremadura Spur actinomycetes Qemistree. Outer ring displays the relative abundance of metabolites stratified by the genera of the strains. Genera colors are according to the legend. Inner ring represents the structural relationships between metabolites establishing a chemical class hierarchy. The presented structures are spectral reference library matches obtained from the GNPS.

Mass spectrometry data of the Estremadura Spur actinomycete extracts was analyzed by Qemistree (Figure 8), a new GNPS analysis tool recently developed by Dorrestein et al. [73] in which molecular relationships are represented as a chemical tree based on the hierarchical organization of molecular fingerprints predicted from fragmentation spectra. While the molecular network visualizes closely related molecular families, Qemistree uses fragmentation trees and supervised machine learning from CSI:FingerID [74] to calculate all pairwise chemical relationships, and then visualizes it in the context of sample metadata and molecular annotations that account for the inference of chemical relationships in a dataset. Qemistree representation of the MS/MS data allowed in-silico matching, using CSI:Finger ID (level 3, according to MSI [50]), with fatty acids to the *Micromonospora* and *Streptomyces* strains, and macrolactams and prenol lipids to *Streptomyces* (Figure 8).

Unlike the crude extracts from the Madeira Archipelago and other locations previously studied by our group [23], 66.7% (*n* = 84) of the Estremadura Spur crude extracts have oil consistency. Qemistree reveals the presence of fatty acids, suggesting that these strains have evolved to produce these chemical compounds in a higher abundance than strains from other locations. Based on these results, a detailed investigation of the Estremadura Spur fatty acids by GC-MS, and the evaluation of their biotechnological potential, is currently ongoing in our lab. From the geological point of view, these results suggest that the Estremadura Spur pockmarks are dormant and not inactive, as initially presumed. Sulfate-reducing bacteria (SRB) involved in the anaerobic oxidation of methane (AOM) produce characteristic fatty acids. These lipidic biomarkers can provide evidence for the role played by archaea and SRB in AOM. Our results suggest that similar traits may occur with actinomycetes [75]. The Qemistree representation also showed that the majority of the chemical classes seemed to act as chemotaxonomic markers, as they presented a genera-specific distribution.

### 2.6. The Evaluation of the Actinomycetes Biotechnological Potential

In order to assess the biotechnological potential of the marine-derived actinomycetes from the Estremadura Spur pockmarks, 84 crude extracts were tested for their antimicrobial activity against three human pathogenic bacteria: methicillin-resistant *Staphylococcus aureus* (MRSA, COL); methicillin-susceptible *Staphylococcus aureus* (MSSA, NCTC 8325) and *Escherichia coli* (K12) (Table 3); the human pathogenic yeast *Cryptococcus neoformans*; and the dermatophytic fungus *Trichophyton rubrum* (Table 4). The anticancer activity and general toxicity of the extracts were assessed against the human colon carcinoma cell line HCT-116 and the non-cancerous human keratinocyte cell line HaCaT, respectively. Finally, the extracts were evaluated for their cellular antioxidant activities using a 2′7′-dichlorofluorescin diacetate (DCFH-DA) radical on the human keratinocytes (HaCaT) (Table 5).

### 2.7. Antibacterial Activity Evaluation

Twenty-six actinomycetes crude extracts revealed antibacterial activity, although several showed residual activity; 15 against MRSA, 14 against MSSA, and 14 against *E. coli* (Table 3).

As expected, most extracts that showed growth inhibition activity against the MRSA strain also showed activity against the MSSA strain (12 out of 17 active extracts, 70.6%). Interestingly, most extracts that exhibited activity against the Gram-positive bacterium *S. aureus* did not inhibit the growth of the Gram-negative bacterium *E. coli*; namely, 12 out of the 17 extracts (70.6%). A reverse behavior was also observed, i.e., with the extracts with anti-*E. coli* activity, 9 out of the 14 extracts (64.3%) were inactive against *S. aureus*. These observations suggest that the compounds present in the extracts may predominantly target the bacterial cell wall. The most active extracts were *S. aculeolatus* (MT830758) that inhibited, equipotently, both MRSA and MSSA strains (MIC value 3.9 µg/mL) and *S. ovatisporus* (MT830803), which had four-fold higher activity against MSSA (MIC 7.8 µg/mL) than MRSA (MIC 31.3 µg/mL). The remaining extracts with *S. aureus* inhibitory activity had MIC values ranging from 31.3 to 250 µg/mL (Table 3). Strikingly good activity was observed with the *Micromonospora matsumotoense* (MT830774) extract, which demonstrated the highest activity against the Gram-negative strain *E. coli* (MIC 1.9 µg/mL), while all other extracts, as mentioned above, had marginal potency, with MIC values ranging from 125 to 250 µg/mL against this bacterium. Remarkably, the *M. matsumotoense* extract exhibited an MIC value (1.9 µg/mL) lower than the standard compound tetracycline (3.9 µg/mL) (Table 3). Amongst the bioactive strains were the antimycin producing strains, namely, PTE-036, PTE-040, PTE-049, and PTE-064; the etamycin producing strain PTE-054; the ikarugamicin producing strains PTE-018, PTE-033, PTE-034, PTE-054, PTE-059, PTE-063, PTE-064, PTE-065, and PTE-071; the pieridicin producing strains PTE-024, PTE-034, PTE-036, and PTE-054; and the rakicidin producing strains PTE-024 and PTE-065. Nevertheless, the majority of the bioactive strains produced metabolites that have not been found in the spectral libraries in the GNPS, highlighting the prospects for the future isolation of novel antibacterial compounds.

### 2.8. Antifungal and Antiyeast Activity Evaluations

Three extracts showed moderate activity against the pathogenic fungus *Trichophyton rubrum* and the yeast *Cryptococcus neoformans*. As shown in Table 4, *S. sampsoni* and *S. intermedius* extracts demonstrated activity against the yeast *C. neoformans* with IC_50_ values of 12.2 and 11.4 µg/mL. The yeast *C. neoformans* is linked mainly to lung infections, meningitis, and encephalitis particularly in immunocompromised patients [76,77]. A low anti-dermatophytic potential was exerted against *Trichophyton rubrum* by the extracts of *S.* *chumphonensis* and *S. sampsoni* (IC_50_ values of 59.6 and 58.9 µg/mL, respectively). The dermatophyte fungus, *T. rubrum*, also found in the skin microbiome, is responsible for athlete’s foot, nail infections, ringworm and jock itch [78].

### 2.9. Anticancer and Cytotoxicity Evaluations

The Estremadura Spur actinomycetes did not exert anticancer activity against human colon carcinoma (HCT-116) cells at the highest test concentrations (100 µg/mL). Out of 84 extracts, 11 showed low activities against the HCT-116 cells with 20% to 50% inhibition rates at 100 µg/mL, hence, the IC_50_ values were not determined. The extracts also lacked toxicity against the human keratinocyte HaCaT cells. Despite the annotation of surugamides and rakicidins with known anticancer activities [51], our anticancer activity results are not very potent, which might be due to the low concentrations of these metabolites in the extracts. Similarly, surugamides were detected mainly in noncytotoxic *Streptomyces* extracts from Rocas Atoll [55].

### 2.10. Antioxidant Activity Evaluations

Four extracts belonging to the *Streptomyces* species showed significant antioxidant activities, with IC_50_ values ranging from 11.2 to 69.2 µg/mL against the 2′7′-dichlorofluorescin diacetate (DCFH-DA) radical on HaCaT cells. Notably, the *S. aculeolatus* extract exhibited an IC_50_ value (5.0 µg/mL) in the same magnitude as the standard compound luteolin (IC_50_ 4.0 µg/mL) (Table 5).

## 3. Materials and Methods

### 3.1. Marine Sediment Collection

The sediment samples were collected from the Estremadura Spur pockmark field using a Smith McIntyre Grab between 31 May and 5 June 2017. Surface sediment (0–1 cm) was picked at 14 stations (Table S1) situated at the center of randomly selected pockmarks and the open sediments in proximity to the pockmarks (Figure 1). The distance between stations varied up to 10 km. After collection, the samples were placed in labelled sterile bags (Nasco whirl-pack) and stored at −20 °C until further processing.

### 3.2. Marine Sediment Inoculation

All the sediments collected were inoculated onto the surface of the three following agar media: medium 1 (A1), 18 g of agar, 10 g of starch, 4 g of yeast extract, 2 g of peptone; medium 2 (½ A1), 18 g of agar, 5 g of starch, 2 g of yeast extract, 1 g of peptone; and medium 3 (SWA), 18 g of agar. All media were prepared with filtered seawater and deionized (DI) water in the proportion of 75:25 (*v*/*v*), supplemented with the antifungal agent cycloheximide (100 µg·mL^−1^). The inoculation was performed using two methods, heat-shock and drying, as described [23,79]. Briefly, in the first method, approximately 0.5 g of wet sediment was diluted with 2 mL of sterile seawater (SSW). Then, the diluted sample was allowed to settle for two minutes, heated to 55 °C for 6 min, and then 100 µL of the resulting solution was spread on the agar plates. In the second method, the sediments were dried overnight in a laminar flow hood. After this period, an autoclaved plug 1 to 2 cm in diameter was pressed onto the sediment samples and then repeatedly spread onto the surface of the agar plates in a clockwise direction, creating a serial dilution effect. These techniques were performed to reduce the isolation of Gram-negative bacteria and fungi, and also to enrich the slow-growing, spore-forming actinomycetes.

### 3.3. Actinomycete Isolation and Quantification

In order to monitor actinomycete growth, all the previously inoculated agar plates were incubated at 25 °C and monitored periodically over 6 months. The unicellular bacteria were recognized by the presence of filamentous hyphae and/or by the formation of tough, leathery colonies that adhered to the agar surface [80]. Therefore, for this study, only the mycelium-forming bacteria belonging to the order Actinomycetales were included. For every plate that yielded actinomycete colonies, the total number of colonies observed was counted, and representatives of all morphotypes were obtained in pure culture by their repeated transfer to A1 medium agar plates from a single colony. The pure strains isolated were grouped based on the presence or absence of aerial mycelium, the colony size, morphology, the colony color, spore appearance, diffusible pigment production, and the presence or absence of aerial hyphae and the effects of seawater on growth. Eighty-five pure actinomycetes isolates were obtained, which were subsequently cultured in a liquid medium (A1 without agar) and cryopreserved in 10% (*v*/*v*) glycerol at −80 °C.

### 3.4. Seawater Requirement for Growth

The requirement of seawater for the growth of the 85 actinomycetes was assessed, as described [14,23]. This was accomplished by replacing seawater with DI water in the culture medium and observing the effects on growth through visual monitoring for 6 months. Cells from a well-defined colony were inoculated onto medium A1DI plates (A1 prepared with DI water). Plates were incubated at 25 °C and, if no growth was observed on the A1DI plate, that strain was determined to require seawater for growth. The seawater obligate strains were replated three times for confirmation.

### 3.5. DNA Extraction, 16S rRNA Gene Amplification, and Sequencing

All strains were inoculated in 4 mL of medium A1 and incubated at 25 °C for 3 to 7 days with agitation (200 rpm). Total DNA was extracted using the Wizard Genomic DNA Purification Kit (Promega, Madison, WI, USA) as described by the manufacturer, with some adjustments, as described [23,36,80]. The 16S rRNA gene was amplified using the primers FC27 (5′-AGAGTTTGATCCTGGCTCAG-3′), RC1492 (5′-TACGGCTACCTTGTTACGACTT-3′) [14,80,81], and NZYTaq II DNA polymerase (NZYtech, Lisbon, Portugal). The PCR products were purified using the NZYGelpure PCR clean-up kit (NZYtech) and were sequenced by the Sanger method using the primers described above at STAB VIDA (https://www.stabvida.com, accessed on 20 July 2021), where the ABI BigDye^®^ Terminator v3.1 Cycle Sequencing Kit was used.

### 3.6. Taxonomic Classification

The 16S rRNA gene sequencing chromatograms were reviewed and edited with 4Peaks 1.8 (Nucleobytes B.V.). Consensus sequences for each forward/reverse pair were created in Jalview 2.11.1.0 [82] using the multi-sequence aligner MAFFT [83]. Each consensus sequence was compared to the EzTaxon database [84] with the 16S-based ID tool (on 10 July 2020), and to the NCBI (GenBank) rRNA/ITS database with the BLASTn algorithm [85] (on 16 July 2020). When the best hits of the two database searches corresponded to the same species, considering a threshold of 99% sequence identity, the isolate was taxonomically identified. If both methods differed in their species classification, a further phylogenetic analysis was used to clarify the isolate’s species description (see the next section). For that, the representative 16S rRNA gene sequence of each of the species obtained in the best hits of both database searches were retrieved from NCBI (Table S2).

### 3.7. Phylogenetic Analyses

For the phylogenetic analysis, the consensus sequence of each isolate and the representative sequences of each of the species identified from the best hits of the searches in the EzTaxon and NCBI databases (Table S2) were aligned with the online version of MAFFT (https://mafft.cbrc.jp/alignment/server/, accessed on 23 September 2021) using the G-INS-i interactive refinement method. The multi-sequence alignment was trimmed at 5′ and 3′ to compensate for different sequence sizes, leaving sequences of 1000 bp for further analyses. MEGAX 10.1.7 software (http://www.megasoftware.net, accessed on 23 September 2021) [86,87] was used to infer the best DNA substitution model for each trimmed multi-sequence alignment. The model with the lowest BIC (Bayesian Information Criterion) score was used for further phylogenetic analyses [88]. A maximum likelihood tree reconstruction of the trimmed multi-sequence alignment was performed with the same program. Bootstrap coefficients were calculated for 1000 replicates. *Bacillus methanolicus* PB1(AFEU01000002) was used as outgroup.

### 3.8. Operational Taxonomic Units (OTUs) Groupings

Operational taxonomic units (OTUs) were defined for the 16S rRNA gene sequences of the cultivated actinomycetes using the tools available in MOTHUR v2.13.3 (http://www.mothur.org/, accessed on 25 July 2021) [89] using the default parameters, except when mentioned otherwise. Briefly, these sequences were aligned to the Greengenes-formatted database (available at https://mothur.org/wiki/alignment_database/ accessed on 25 July 2021) using the align.seqs function. The summary.seqs function was used to obtain the summary statistics of the sequence collection. All the sequences reported as problematic were then removed with the screen.seqs function, and the alignment was trimmed with filter.seqs. A phylip-formatted distance matrix of the trimmed multi-sequence alignment was obtained with dist.seqs. Finally, the cluster.classic function, considering an average neighbor algorithm [89], was used to create the OTUs at a 97% identity threshold. A representative sequence for each OTU was obtained with the get.oturep command.

### 3.9. Rarefaction and the Diversity Estimation Analysis

The rarefaction analysis and the diversity estimation of the actinomycete sequences were calculated using a nonparametric richness estimator, Chao 1 in MOTHUR using the rarefaction.single command, as described by [90,91]. This command generated intra-sample rarefaction curves using a resampling without replacement approach. Rarefaction curves provide a way of comparing the richness observed in different samples [89]. The Shannon index diversity is commonly used in bacterial diversity measurements based on operational taxonomic units and provides more inference about the community composition than simple species richness or evenness does [92].

### 3.10. Novel OTU Determination

A representative strain of each OTU was compared to type strains present in the EzTaxon database [84] for 16S rRNA sequence similarity. As described in [23], if a given sequence had less than a 98% sequence similarity to the nearest type strain, all the strains in that OTU have to be compared to the type strain in order to determine if the whole OTU could be considered novel.

### 3.11. Crude Extract Preparation

The 85 isolated actinomycete strains were cultured under identical growth conditions as described [23]. Seed cultures of 20 mL in 100 mL flasks with the medium A1 were grown for 7 days prior to transfer. Each strain was inoculated in 1L of medium A1 with shaking at 220 rpm at 30 °C for 7 days. The culture broth was extracted with EtOAc and the organic layer was concentrated to dryness under vacuum. PTE-008 *Actinopolymorpha cephalotaxi* was the only strain that did not grow in enough amounts to produce a crude extract under the used conditions. The crude extracts were dissolved in MeOH at 2 mg·mL^−1^ concentration for LC-MS/MS analyses and dissolved in DMSO at 10 mg·mL^−1^ concentration for bioactivity screening.

### 3.12. Untargeted Metabolomic Fingerprint by LC-MS/MS

All samples except the PTE-008 crude extract were injected into a Vanquish UHPLC system coupled to a Q-Exactive orbitrap mass spectrometer (Thermo Fisher Scientific, Bremen, Germany), according to [93]. The chromatographic separation occurred in a C18 porous core column (Kinetex C18, 50 × 2 mm, 1.8 um particle size, 100 A pore size, Phenomenex, Torrance, CA, USA) kept at 30 °C. The mobile phase consisted of solvent A, H_2_O + 0.1% formic acid (FA), and solvent B, acetonitrile (ACN) + 0.1% FA, with a flow rate of 0.150 mL/min. Five µL of the sample was injected and eluted with the following linear gradients: 0.0–4.0 min at 5–50% of B, 4.0–5.0 min at 50–99% of B, followed by a 2 min washout phase at 99% B and a 3 min re-equilibration phase at 5% B (method 1), or 0–0.5 min at 5% of B, 0.5–9 min at 5–100% of B, followed by a 2 min washout phase at 99% B and a 5 min re-equilibration phase at 5% B. MS/MS data was acquired with data dependent acquisition (DDA) performed in the positive mode.

Electrospray ionization (ESI) parameters were set to 53 L/min sheath gas flow, 14 L/min auxiliary gas flow, 0 L/min sweep gas flow, and 400 °C auxiliary gas temperature. The spray voltage was set to 3.5 kV, and the inlet capillary at 320 °C and 50 V S lens levels were applied. The MS scan range was set to 200–2000 *m*/*z* with a resolution at *m*/*z* 200 (R*_m_*_/*z* 200_) of 35,000 with one micro scan. The maximum ion injection time was set to 100 ms with an automated gain control (AGC) target of 5E5. Up to 5 MS/MS spectra per MS1 survey scan were recorded in DDA mode with R*_m_*_/*z*_ 200 of 17,500 with one micro-scan. The maximum ion injection time for MS/MS scans was set to 100 ms with an AGC target of 5E5 ions. The MS/MS precursor isolation window was set to *m/z* 1. The normalized collision energy was set to a stepwise increase from 20%, to 30%, to 40% with z = 1 as the default charge state. MS/MS scans were triggered at the apex of the chromatographic peaks within 2 to 15 s from their first occurrence. Dynamic precursor exclusion was set to 5 s. Ions with unassigned charge states were excluded from MS/MS acquisition as well as isotope peaks.

### 3.13. Classical Molecular Networks

A molecular network was created using the online workflow (https://ccms-ucsd.github.io/GNPSDocumentation/, accessed on 23 September 2021) on the GNPS website (http://gnps.ucsd.edu accessed on 23 September 2021). The data was filtered by removing all MS/MS fragment ions within +/− 17 Da of the precursor *m*/*z*. MS/MS spectra were window filtered by choosing only the top six fragment ions in the +/− 50 Da window throughout the spectrum. The precursor ion mass tolerance was set to 0.02 Da with a MS/MS fragment ion tolerance of 0.02 Da. A network was then created where the edges were filtered to have a cosine score above 0.65 and more than five matched peaks. The 0.65 threshold was used for a more sensitive connectivity of the network. Furthermore, the edges between two nodes were kept in the network if each of the nodes appeared in each other’s respective top ten most similar nodes. Finally, the maximum size of a molecular family was set to 100, and the lowest scoring edges were removed from molecular families until the molecular family size was below this threshold. The spectra in the network were then searched against the GNPS’ spectral libraries. The library spectra were filtered in the same manner as the input data. All matches kept between the network spectra and the library spectra were required to have a score above 0.65 and at least five matched peaks.

### 3.14. The LC-MS/MS Data Process for Qemistree

The raw data was converted to a .mzXML format using MSConvert (part of ProteoWizard version 2.1.x, GitHub, San Francisco, CA, USA) [94,95]. For feature detection, the data were processed with MZmine2 2.51 [96,97] using the following parameters: a MS1 noise level of 8.0 × 10^5^, and a MS2 noise level of 1.0 × 10^3^. Chromatograms were built with the ADAP chromatogram builder [98] module with the following parameters: “Min group size in # of scans” = 2; “Group intensity threshold” = 8.0 × 10^5^, “Min highest intensity” = 5.0 × 10^5^; and “maximum *m*/*z* tolerance” = 10 ppm. The built chromatograms were deconvoluted using the ADAP (Wavelets) module, and the following parameters were used: a “S/N threshold” of 10; a “min feature height” of 8.5 × 10^5^; a “coefficient/area threshold” of 30; a “Peak duration range” of 0.01–2.0 min; and a “RT wavelet range” of 0.01–0.2 s. The fragmentation spectra were paired to the deconvoluted peaks using 0.05 Da and 0.2 min windows. The LC-MS features (isotopologues, adducts, and in-source fragments) were annotated using the peak grouping module with the following parameters: “deisotope” = true, *m*/*z* tolerance at 10 ppm, retention time tolerance at 0.2 min, and a max. charge of 3. The features were aligned with the join aligner module using the following parameters: 7 ppm tolerance, weight for *m*/*z* of 75.0, a retention time tolerance of 0.2 min, and a weight for RT of 25.0. Finally, the feature table was exported as a .CSV and the spectral data as a .MGF and a SIRIUS file. Using the GNPS export module for further processing, only MS^1^ peaks that had a MS^2^ spectrum were considered (GNPS filter).

The .MGF, the features intensity (.csv), and the SIRIUS files were uploaded to the GNPS [47] and analyzed with the feature-based molecular network workflow (https://ccms-ucsd.github.io/GNPSDocumentation/featurebasedmolecularnetworking/ accessed on 23 September 2021). Spectral library matching was performed against the public spectral library. Molecular features in the form of MS/MS spectra were putatively identified using MS2-based spectral library matches based on the cosine score similarity (above 0.7) and a minimum number of shared peaks (6). The parameters and results can be consulted at the following address: https://gnps.ucsd.edu/ProteoSAFe/status.jsp?task=401f1ece67dc449f99cdc43a7308b543 accessed on 23 September 2021. The results were subsequently visualized in Cytoscape 3.6 [99]. The MN was colored by genus: *Actinomadura* (dark green), *Micromonospora* (light green), *Nocardiopsis* (yellow), *Saccharomonospora* (orange), *Saccharopolyspora* (pink), *Streptomyces* (dark blue), and *Verrucosispora* (light blue).

The Qemistree [73], a computing tree-based approach, was generated in the GNPS and the resulting chemical hierarchy was explored in iTOL [100].

### 3.15. Bacterial Growth Inhibition Assays

The antibacterial activity of the crude extracts was evaluated by performing growth inhibition assays for two strains of the Gram-positive human opportunistic pathogen *S. aureus*, namely, the methicillin-resistant *S. aureus* strain COL (MRSA) and the methicillin-sensitive *S. aureus* strain NCTC8325–4 (MSSA), as well as against the Gram-negative bacterium *Escherichia* *coli* strain K12. The *S. aureus* strains were grown in tryptic soy broth (TSB; Becton Dickinson, Germany), and *E. coli* in Lysogeny broth (NZYtech), at 37 °C.

The assays were performed in 96-well polystyrene flat bottom microplates (Nunclon Delta Surface, Thermo Scientific, Roskilde, Denmark). Bacterial overnight cultures were diluted to an optical density (OD600nm) of 0.04–0.06 and were incubated statically in the presence of different concentrations of each crude extract, solubilized in DMSO. All cultures were two-fold serially diluted, resulting in final concentrations of the extracts ranging from 250 to 1.95 μg/mL. After 24 h of incubation at 37 °C, the minimal inhibitory concentration (MIC) value was determined by visual inspection and by a spectrophotometric analysis. The active crude extracts were re-tested for results confirmation. The resulting values were compared with the positive control (vancomycin for MRSA and MSSA, and tetracycline for *E. coli* K12), a DMSO solvent control, and a negative control (no extract) on the same plate. All assays were performed in triplicate.

### 3.16. Antiyeast and Antifungal Assays

Crude extracts were tested against the human pathogen yeast *Cryptococcus neoformans* (DSM 6973, Leibniz Institute DSMZ-German Collection of Microorganisms and Cell Cultures, Braunschweig, Germany) and the dermatophyte *Trichophyton rubrum* (I/95, patient isolate from university clinic of dermatology, Prof. Brasch, Kiel, Germany).

*C. neoformans* was cultivated in a M186 medium (1% glucose, 0.5% peptone from soymeal, 0.3% malt extract, and a 0.3% yeast extract). An overnight culture of *C. neoformans* (28 °C and 160 rpm) was prepared and diluted to an optical density (600 nm) of 0.03. The cultivation of *T. rubrum* took place on a GPY agar (0.1% glucose, 0.05% peptone, 0.01% yeast extract, and 1.5% agar, at a pH of 7.2–7.4) for two weeks. The spores of the fungus were removed and dissolved in a liquid M186 medium (1% glucose, 0.5% soy peptone, 0.3 yeast extract, and 0.3% malt extract) and the concentration was determined with a counting cell chamber. The spore solution was diluted to a final concentration of 5 × 10^4^ spores/mL in the M186 medium. The extracts (10 mg/mL DMSO stock solution) were transferred into a 96-well microtiter plate in duplicate and 200 μL of the cell suspension cultures were added to each well. The final assay concentration of samples was 100 μg/mL. The inoculated microplates were incubated for 7 h at 28 °C and at 200 rpm for *C. neoformans*, and 72 h at 28 °C and at 120 rpm for *T. rubrum*. To detect the inhibitory effect of the extracts against *C. neoformans* and *T. rubrum,* the optical density at 600 nm after the incubation time was recorded using the microplate reader (Tecan Infinite 200). The resulting values were compared with positive controls (amphotericin B for *C. neoformans* and clotrimazol for *T. rubrum*) and a negative control (no extract) on the same plate. For IC_50_ determination, a dilution series was prepared and the IC_50_ value was calculated using Excel as the concentration that showed a 50% inhibition of viability on the basis of a negative control.

### 3.17. Cytotoxic and Anticancer Assays

The actinomycete crude extracts were tested in vitro against two human cell lines: the colon carcinoma cell line HCT-116 (DSMZ, Braunschweig, Germany) and the non-cancerous human keratinocyte line HaCaT (CLS, Eppelheim, Germany) at a final concentration of 100 μg/mL. HaCaT cells were cultivated in a RPMI medium (Life Technologies, Darmstadt, Germany) and HCT-116 cells in a DMEM medium (Life Technologies), supplemented with 4.5 g/L D-Glucose and 110 mg/L sodium pyruvate. All media were supplemented with L-Glutamine, 10% fetal bovine serum (FBS, Life Technologies), 100 U/mL penicillin, and 100 mg/mL streptomycin (P/S, Life Technologies). The cultures were maintained at 37 °C under a humidified atmosphere and 5% CO_2_. The cell lines were transferred every 3 or 4 days. For the experimental procedure, the cells were seeded in 96-well-plates at a concentration of 10,000 cells per well. After 24 h of incubation, the medium was removed from the cells and 100 µL of the fresh medium containing the test samples was added. Plates were cultured at 37 °C and 5% CO_2_ for 24 h. Each sample was prepared in triplicate. Doxorubicin (50 μg/mL), as a standard therapeutic drug, was used as positive control and a growth medium was used as negative control. The anticancer activity was evaluated by monitoring the metabolic activity using the CellTiterBlue Cell Viability Assay (Promega, Mannheim, Germany). Briefly, 20 µL of the CellTiterBlue reagent was added to each well and incubated for 2 h. Afterwards, the assay was performed according to the manufacturer’s instructions and measured using the microplate reader Tecan Infinite M200 at an excitation of 560 nm and an emission of 590 nm. The percentage of inhibition was calculated by comparing the positive control and negative control (no extract) on the same plate. A dilution series was prepared and the IC_50_ value was calculated by Excel as the concentration that showed a 50% inhibition of viability on the basis of a negative control (growth medium).

### 3.18. The Cellular Antioxidant Activity (CAA) Assay

The antioxidant activity of the crude extracts against the 2′7′-dichlorofluorescin diacetate (DCFH-DA) radical was evaluated by measuring the cellular antioxidant activity (CAA) on the human keratinocytes HaCaT. HaCaT cells were seeded at a density of 100,000 cells/well and incubated overnight at 37 °C under a humidified atmosphere and 5% CO_2_. Cells were then incubated with DCFH-DA (2′7′-dichlorofluorescin diacetate; Sigma, Burlington, MA, USA) (a final concentration of 25 μM) and the test samples (a final concentration of 100 μg/mL) in duplicate for 1 h. Luteolin (final concentrations of 100 μM) was used as positive control. After incubation, 600 μM of AAPH (2,2′-azobis(2-methylpropionamidine) dihydrochloride, Sigma) was added to all the wells. The plate was immediately placed in the plate reader and the fluorescence was recorded. An excitation of 485 nm and an emission of 520 nm were used. The plate was incubated for 10 min before a second reading. Cells were washed with Hank’s balanced salt solution (HBSS, Life Technologies) between the additions of new reagents. The total reaction volume was 100 μL (where 50 μL was the volume of the test sample). The incubations were at 37 °C in a humidified atmosphere of 5% CO_2_. The resulting values were compared with a positive control and a negative control (no extract) on the same plate. For IC_50_ determination, a dilution series was prepared and the IC_50_ value was calculated by Excel as the concentration that showed a 50% inhibition of viability on the basis of a negative control.

## 4. Conclusions

This study showed the biotechnological potential of the Estremadura Spur pockmarks actinomycete isolates as a source of antimicrobial and antioxidant agents. It led to the isolation and purification of 85 actinomycetes, 18 of which showed prospects of being marine obligate strains comprised of several seawater requiring strains from genera that have not been reported before and that have the ability to produce exclusive molecular families.

Actinomycetes strains, including “marine obligate” strains, revealed antimicrobial activity against Gram-positive and Gram-negative multidrug resistant bacteria, which allows the prioritization of strains for future compound isolation and structure elucidation. Importantly, sea water requiring strains revealed unknown exclusive molecular family clusters, suggesting that they are a source of novel natural products.

High chemical diversity was revealed in our results. Interestingly, this study highlights the phylogenetically related actinomycetes with different biosynthetic capabilities and shows that the distribution of the majority of the chemical classes was genus-specific, suggesting that the produced metabolites can be used as chemotaxonomic markers.

The Estremadura Spur strains showed a propensity to produce fatty acids, which will lead to an in-depth study of these compounds in the future to evaluate their composition and prospective applications. Moreover, these results suggest that the Estremadura Spur pockmarks may be dormant and not inactive as presumed, as actinomycetes from their sediments have a clear carbon source that allows them to produce fatty acid, suggesting that some oil seeping could still be happening in the area. Overall, this study demonstrates that the regions surrounding the Portuguese coast, in particular the singular Estremadura Spur pockmark field, are rich sources of marine actinomycetes with potential applications for biotechnology.

## Figures and Tables

**Table 1:** Genera and number of isolates, OTUs, and species for each genus isolated from the Estremadura Spur, continental Portugal coast marine sediments.

Genus	Number of Isolates	Number of OTUs	Number of Species
*Streptomyces*	35	24	13
*Micromonospora*	30	19	16
*Saccharopolyspora*	6	2	1
*Saccharomonospora*	5	2	1
*Actinomadura*	3	2	2
*Nocardiopsis*	3	1	1
*Actinopolymorpha*	1	1	1
*Stackebrandtia*	1	1	1
*Verrucosispora*	1	1	1
Total	85	53	37

**Table 2:** Diversity and distribution of actinomycetes from the Estremadura Spur, continental Portugal coast, according to the geographic locations. The numbers 1 to 14 in the header correspond to each of the codes of the geographic locations of the sediment collection: 1 (D01PC1), 2 (D01PC3), 3 (PES GR1), 4 (PES GR6), 5 (PES GR9), 6 (PESGR14), 7 (PESGR17), 8 (PESGR20), 9 (PESGR22), 10 (PESGR23), 11 (PESGR24), 12 (PESGR25), 13 (PESGR26), 14 (PESGR27).

Stations	1	2	3	4	5	6	7	8	9	10	11	12	13	14
OTUs	3	1	6	3	1	1	10	14	6	11	1	8	2	2
N° of isolates	5	1	7	4	1	1	16	15	6	16	1	8	2	2
N° of samples	1	1	1	1	1	1	1	1	1	1	1	1	1	1
Isolates/Sample	5	1	7	4	1	1	16	15	6	16	1	8	2	2
Shannon index	0.50	0.00	1.55	0.56	0.00	0.00	1.99	2.25	1.79	1.67	0.00	2.08	0.69	0.69

**Table 3:** Antimicrobial activity of the marine actinomycetes crude extracts against methicillin-resistant *Staphylococcus aureus* (MRSA, strain COL), methicillin-susceptible *Staphylococcus aureus* (MSSA, strain NCTC 8325), and *Escherichia coli* (strain K12). MIC values are in µg/mL. NA—Not Active. — symbol—Not Applicable.

Extract Code	GenBank Accession Number	Species Best Match Identity (%) in the NCBI Database	MRSA	MSSA	*E. coli*
PTE-006	MT830755	*Streptomyces griseochromogenes* (99%)	125	125	NA
PTE-009	MT830758	*Streptomyces aculeolatus* (99%)	3.9	3.9	NA
PTE-010	MT830759	*Streptomyces griseolus* (99%)	250	250	NA
PTE-015	MT830764	*Micromonospora schwarzwaldensis* (100%)	NA	NA	250
PTE-018	MT830767	*Streptomyces xiamenensis* (99%)	250	250	NA
PTE-023	MT830772	*Streptomyces malachitospinus* (100%)	NA	NA	250
PTE-024	MT830773	*Micromonospora chalcea* (100%)	NA	NA	125
PTE-025	MT830774	*Micromonospora matsumotoense* (99%)	62.5	125	1.9
PTE-032	MT830781	*Streptomyces intermedius* (100%)	NA	NA	125
PTE-033	MT830782	*Actinomadura geliboluensis* (99%)	250	NA	NA
PTE-034	MT830783	*Streptomyces chumphonensis* (99%)	62.5	125	NA
PTE-036	MT830785	*Streptomyces gougerotii* (99%)	250	NA	NA
PTE-040	MT830789	*Streptomyces sampsonii* (100%)	250	NA	NA
PTE-042	MT830791	*Streptomyces aculeolatus* (100%)	NA	62.5	NA
PTE-049	MT830798	*Streptomyces intermedius* (99%)	250	62.5	250
PTE-051	MT830800	*Streptomyces sampsonii* (99%)	NA	NA	125
PTE-052	MT830801	*Streptomyces griseolus* (100%)	NA	250	250
PTE-054	MT830803	*Streptomyces ovatisporus* (99%)	31.3	7.8	250
PTE-055	MT830804	*Micromonospora echinospora* (99%)	NA	NA	250
PTE-059	MT830808	*Streptomyces xiamenensis* (99%)	62.5	125	NA
PTE-063	MT830812	*Streptomyces xiamenensis* (99%)	31.3	31.3	NA
PTE-064	MT830813	*Streptomyces sampsonii* (100%)	NA	NA	250
PTE-065	MT830814	*Streptomyces xiamenensis* (99%)	250	250	250
PTE-070	MT830819	*Streptomyces xiamenensis* (99%)	31.3	31.3	NA
PTE-071	MT830820	*Streptomyces intermedius* (99%)	NA	NA	250
PTE-077	MT830826	*Saccharopolyspora gloriosae* (99%)	NA	NA	250
Vancomycin	—	Positive control	1.9	1.9	—
Tetracycline	—	Positive control	—	—	3.9

**Table 4:** Results of actinomycetes crude extracts against the human pathogen yeast *Cryptococcus neoformans* and the dermatophyte *Trichophyton rubrum.* IC_50_ values are in µg/mL. — symbol—Not Applicable.

Strain/Extract Code	GenBank Accession Number	Species	*C. neoformans*	*T. rubrum*
PTE-034	MT830783	*Streptomyces chumphonensis*	>100	59.6 ± 1.4
PTE-040	MT830789	*Streptomyces sampsonii*	12.2 ± 0.2	58.9 ± 0.7
PTE-053	MT830802	*Streptomyces intermedius*	11.4 ± 1.9	>100
Amphotericin B	—	Positive control	0.06 ± 0.00	—
Clotrimazol	—	Positive control	—	0.04 ± 0.00

**Table 5:** Antioxidant activity (CAA) of the extracts on the human keratinocytes HaCaT cell line. IC_50_ values are in µg/mL. — symbol—Not Applicable.

Strain/Extract Code	GenBank Accession Number	Species	HaCaT
PTE-001	MT830750	*Streptomyces gougerotii*	11.2 ± 1.4
PTE-006	MT830755	*Streptomyces griseochromogenes*	69.2 ± 10.3
PTE-009	MT830758	*Streptomyces aculeolatus*	5.0 ± 0.6
PTE-040	MT830789	*Streptomyces sampsonii*	11.8 ± 0.8
Luteolin	—	Positive control	4.0 ± 0.1

## Data Availability

All the nucleotide sequences from the 85 actinomycetes strains were deposited in GenBank under accession numbers MT830750-MT830834 available at https://www.ncbi.nlm.nih.gov/genbank/, accessed on 23 September 2021. All mass spectrometry data and metadata are publicly available in MassIVE MSV000085864.

## Supplementary Materials

The following are available online at https://www.mdpi.com/article/10.3390/md20010021/s1, Table S1: Estremadura Spur pockmark stations of sediment collection. Table S2: NCBI accession number of the representative 16S rRNA gene sequence of each of the species found as best hit from the search in NCBI rRNA/ITS and EzTaxon databases. Table S3: List of all strains isolated from the Estremadura Spur, continental Portugal coast, including their station, habitat, depth, sea water requirement, and accession number from GenBank (AccessID). Figure S1: Maximum likelihood phylogenetic tree of the alignment of the 16S rRNA gene from all (85) actinomycetes isolates from Estremadura Spur, continental Portugal, and the representative sequences of all species retrieved from the GenBank. Figure S2: Maximum likelihood phylogenetic tree of the alignment of the 16S rRNA gene from *Streptomyces* (A) and *Micromonospora* (B) isolates from Estremadura Spur, continental Portugal, and the representative sequences of all species of the respective genus retrieved from the GenBank. Figure S3: Maximum likelihood phylogenetic trees of the alignments of the 16S rRNA gene from *Streptomyces* (A) and *Micromonospora* (B) isolates from Estremadura Spur, continental Portugal, and the representative sequences of all species of the respective genus retrieved from the GenBank. Figure S4: Maximum likelihood phylogenetic trees of the alignments of the 16S rRNA gene from *Streptomyces* (A) and *Micromonospora* (B) isolates retrieved from GenBank. Figure S5: Rarefaction curves for richness (sobs) and estimated richness (chao) using the statistical estimator Chao 1 of isolated marine derived actinomycetes from the Estremadura Spur, continental Portugal. Figure S6: Venn diagram constructed with the ions detected in the crude extracts. Figure S7: Molecular Network for Estremadura Spur actinomycetes strains that require sea water for their growth.

## References

[1] Judd A., Hovland M. (2007). Seabed Fluid Flow: The Impact on Geology, Biology and the Marine Environment.

[2] Haverkamp T.H.A., Hammer Ø., Jakobsen K.S. (2014). Linking geology and microbiology: Inactive pockmarks affect sediment microbial community structure. PLoS ONE.

[3] Duarte D., Magalhaes V.H., Terrinha P., Ribeiro C., Madureira P., Pinheiro L.M., Benazzouz O., Kim J.-H., Duarte H. (2017). Identification and characterization of fluid escape structures (pockmarks) in the Estremadura Spur, west Iberian margin. Mar. Pet. Geol..

[4] Blunt J.W., Copp B.R., Hu W.-P., Munro M.H.G., Northcote P.T., Prinsep M.R. (2007). Marine natural products. Nat. Prod. Rep..

[5] Tanaka Y., Omura S. (1990). Metabolism and products of actinomycetes: An introduction. Actinomycetologica.

[6] Sivakumar K., Sahu M.K., Thangaradjou T., Kannan L. (2007). Research on marine Actinobacteria in India. Indian J. Microbiol..

[7] Takizawa M., Colwell R.R., Hill R.T. (1993). Isolation and diversity of actinomycetes in the Chesapeake Bay. Appl. Environ. Microbiol..

[8] Mast Y., Stegmann E. (2019). Actinomycetes: The antibiotics producers. Antibiotics.

[9] Goodfellow M., O’Donnell A.G., Goodfellow M., O’donnell A. (1993). Roots of bacterial systematic. Handbook of New Bacterial Systematics.

[10] Stach J.E.M., Maldonado L.A., Ward A.C., Goodfellow M., Bull A.T. (2003). New primers for the class Actinobacteria: Application to marine and terrestrial environments. Environ. Microbiol..

[11] Magarvey N.A., Keller J.M., Bernan V., Dworkin M., Sherman D.H. (2004). Isolation and characterization of novel marine-derived actinomycete taxa rich in bioactive metabolites. Appl. Environ. Microbiol..

[12] Bull A.T., Stach J.E.M., Ward A.C., Goodfellow M. (2005). Marine Actinobacteria: Perspectives, challenges, future directions. Antonie Van Leeuwenhoek.

[13] Fiedler H.-P., Bruntner C., Bull A.T., Ward A.C., Goodfellow M., Potterat O., Puder C., Mihm G. (2005). Marine actinomycetes as a source of novel secondary metabolites. Antonie Van Leeuwenhoek.

[14] Jensen P.R., Gontang E., Mafnas C., Mincer T.J., Fenical W. (2005). Culturable marine actinomycete diversity from tropical Pacific Ocean sediments. Environ. Microbiol..

[15] Feling R.H., Buchanan G.O., Mincer T.J., Kauffman C.A., Jensen P.R., Fenical W. (2003). Salinosporamide A: A highly cytotoxic proteasome inhibitor from a novel microbial source, a marine bacterium of the new genus *Salinospora*. Angew. Chem. Int. Ed..

[16] Jimenez P.C., Wilke D.V., Branco P.C., Bauermeister A., Rezende-Teixeira P., Gaudêncio S.P., Costa-Lotufo L.V. (2019). Enriching cancer pharmacology with drugs of marine origin. Br. J. Pharm..

[17] Barreca M., Span V., Montalbano A., Cueto M., Marrero A.R.D., Deniz I., Erdogan A., Bilela L.L., Moulin C., Taffin-de-Guivenchy E. (2020). Marine anticancer agents: An overview with a particular focus on their chemical classes. Mar. Drugs.

[18] Schneemann I., Nagel K., Kajahn I., Labes A., Wiese J., Imhoff J.F. (2010). Comprehensive investigation of marine Actinobacteria associated with the sponge *Halichondria panicea*. Appl Environ. Microbiol..

[19] Betancur L.A., Naranjo-Gaybor S.J., Vinchira-Villarraga D.M., Moreno-Sarmiento N.C., Maldonado L.A., Suarez-Moreno Z.R., Acosta-González A., Padilla-Gonzalez G.F., Puyana M., Castellanos L. (2017). Marine Actinobacteria as a source of compounds for phytopathogen control: An integrative metabolic-profiling/bioactivity and taxonomical approach. PLoS ONE.

[20] Kavitha A., Savithri H.S. (2017). Biological significance of marine Actinobacteria of east coast of Andhra Pradesh, India. Front. Microbiol..

[21] Parera-Valadez Y., Yam-Puc A., López-Aguiar L.K., Borges-Argáez R., Figueroa-Saldivar M.A., Cáceres-Farfán M., Márquez-Velázquez N.A., Prieto-Davó A. (2019). Ecological strategies behind the selection of cultivable actinomycete strains from the Yucatan Peninsula for the discovery of secondary metabolites with antibiotic activity. Microb. Ecol..

[22] Kashfi R., Kelsey C., Gang D.J., Call D.R., Gang D.R. (2020). Metabolomic diversity and identification of antibacterial activities of bacteria isolated from marine sediments in Hawai’i and Puerto Rico. Front. Mol. Biosci..

[23] Prieto-Davó A., Dias T., Gomes S.E., Rodrigues S., Parera-Valadez Y., Borralho P.M., Pereira F., Rodrigues C.M.P., Santos-Sanches I., Gaudêncio S.P. (2016). The Madeira Archipelago as a significant source of marine-serived actinomycete diversity with anticancer and antimicrobial potential. Front. Microbiol..

[24] Bauermeister A., Velasco-Alzate K., Dias T., Macedo H., Ferreira E.G., Jimenez P.C., Lotufo T.M.C., Lopes N.P., Gaudêncio S.P., Costa-Lotufo L.V. (2018). Metabolomic fingerprinting of *Salinispora* from Atlantic oceanic islands. Front. Microbiol..

[25] Bauermeister A., Pereira F., Grilo I.R., Godinho C.C., Paulino M., Almeida V., Gobbo-Neto L., Prieto-Davó A., Sobral R.G., Lopes N.P. (2019). Intra-clade metabolomic profiling of MAR4 *Streptomyces* from the Macaronesia Atlantic region reveals a source of anti-biofilm metabolites. Environ. Microbiol..

[26] Prieto-Davó A., Fenical W., Jensen P.R. (2008). Comparative actinomycete diversity in marine sediments. Aquat. Microb. Ecol..

[27] Hughes C.C., Prieto-Davó A., Jensen P.R., Fenical W. (2008). The Marinopyrroles, antibiotics of an unprecedented structure class from a marine *Streptomyces* sp.. Org. Lett..

[28] Becerril-Espinosa A., Freel K.C., Jensen P.R., Soria-Mercado I.E. (2013). Marine Actinobacteria from the Gulf of California: Diversity, abundance and secondary metabolite biosynthetic potential. Antonie Van Leeuwenhoek.

[29] Letzel A.-C., Li J., Amos G.C.A., Millán-Aguiñaga N., Ginigini J., Abdelmohsen U.R., Gaudêncio S.P., Ziemert N., Moore B.S., Jensen P.R. (2017). Genomic insights into specialized metabolism in the marine actinomycete *Salinispora*. Environ. Microbiol..

[30] Tsujibo H., Kubota T., Yamamoto M., Miyamoto K., Inamori Y. (2003). Characterization of chitinase genes from an alkaliphilic actinomycete, *Nocardiopsis prasina* OPC-131. Appl. Environ. Microbiol..

[31] Farrell D., Webb H., Johnston M.A., Poulsen T.A., O’Meara F., Christensen L.L.H., Beier L., Borchert T.V., Nielsen J.E. (2012). Toward fast determination of protein stability maps: Experimental and theoretical analysis of mutants of a *Nocardiopsis prasina* serine protease. Biochemistry.

[32] Yuan L.-J., Zhang Y.-Q., Yu L.-Y., Sun C.-H., Wei Y.-Z., Liu H.-Y., Li W.-J., Zhang Y.-Q. (2010). *Actinopolymorpha cephalotaxi* sp. nov., a novel actinomycete isolated from rhizosphere soil of the plant *Cephalotaxus fortunei*. Int. J. Syst. Evol. Microbiol..

[33] Xiong Z.-J., Miao C.-P., Zheng Y.-K., Liu K., Li W.-J., Liu W.-H., Xu L.-H., Zhao L.-X. (2015). *Stackebrandtia endophytica* sp. nov., an actinobacterium isolated from *Tripterygium wilfordii*. Int. J. Syst. Evol. Microbiol..

[34] Liu M.-J., Jin C.-Z., Park D.-J., Asem M.D., Xiao M., Salam N., Li W.-J., Kim C.-J. (2018). *Stackebrandtia soli* sp. nov., a novel actinobacterium isolated from a soil sample. Int. J. Syst. Evol. Microbiol..

[35] Farnaes L., Coufal N.G., Kauffman C.A., Rheingold A.L., DiPasquale A.G., Jensen P.R., Fenical W. (2014). Napyradiomycin derivatives, produced by a marine-derived actinomycete, illustrate cytotoxicity by induction of apoptosis. J. Nat. Prod..

[36] Pereira F., Almeida J.R., Paulino M., Grilo R.I., Macedo H., Cunha I., Sobral R.G., Vasconcelos V., Gaudêncio S.P. (2020). Antifouling napyradiomycins from marine-derived actinomycetes *Streptomyces* aculeolatus. Mar. Drugs.

[37] Taddei A., Rodríguez M.J., Márquez-Vilchez E., Castelli C. (2006). Isolation and identification of *Streptomyces* spp. from Venezuelan soils: Morphological and biochemical studies. I. Microbiol. Res..

[38] Gao R., Liu C., Zhao J., Jia F., Yu C., Yang L., Wang X., Xiang W. (2014). *Micromonospora jinlongensis* sp. nov., isolated from muddy soil in China and emended description of the genus *Micromonospora*. Antonie Van Leeuwenhoek.

[39] Shen Y., Zhang Y., Liu C., Wang X., Zhao J., Jia F., Yang L., Yang D., Xiang W. (2014). *Micromonospora zeae* sp. nov., a novel endophytic actinomycete isolated from corn root (*Zea mays* L.). J. Antibiot..

[40] Zhao S., Liu C., Zheng W., Ma Z., Cao T., Zhao J., Yan K., Xiang W., Wang X. (2017). *Micromonospora parathelypteridis* sp. nov., an endophytic actinomycete with antifungal activity isolated from the root of *Parathelypteris beddomei* (Bak.) Ching. Int. J. Syst. Evol. Microbiol..

[41] Zhao J., Guo L., He H., Liu C., Zhang Y., Li C., Wang X., Xiang W. (2014). *Micromonospora taraxaci* sp. nov., a novel endophytic actinomycete isolated from dandelion root (*Taraxacum mongolicum* Hand.-Mazz.). Antonie Van Leeuwenhoek.

[42] Riesco R., Carro L., Roman-Ponce B., Prieto C., Blom J., Klenk H.P., Normand P., Trujillo M.E. (2018). Defining the species *Micromonospora saelicesensis* and *Micromonospora noduli* under the framework of genomics. Front. Microbiol..

[43] Chao A., Chiu C.-H., Balakrishnan N., Colton T., Everitt W., Piegorsch B., Ruggeri F., Teugels J.L. (2016). Species Richness: Estimation and Comparison.

[44] Jensen P.R., Williams P.G., Dong-Chan O., Zeigler L., Fenical W. (2007). Species-specific secondary metabolite production in marine actinomycetes of the genus *Salinispora*. Appl. Environ. Microbiol..

[45] Román-Ponce B., Millán-Aguiñaga N., Guillen-Matus D., Chase A.B., Ginigini J.G.M., Soapi K., Feussner K.D., Jensen P.R., Trujillo M.E. (2020). Six novel species of the obligate marine actinobacterium *Salinispora*, *Salinispora cortesiana* sp. nov., *Salinispora fenicalii* sp. nov., *Salinispora goodfellowii* sp. nov., *Salinispora mooreana* sp. nov., *Salinispora oceanensis* sp. nov. and *Salinispora vitien*. Int. J. Syst. Evol. Microbiol..

[46] Gaudêncio S.P., Pereira F. (2015). Dereplication: Racing to speed up the natural products discovery process. Nat. Prod. Rep..

[47] Wang M., Carver J.J., Phelan V.V., Sanchez L.M., Garg N., Peng Y., Nguyen D.D., Watrous J., Kapono C.A., Luzzatto-Knaan T. (2016). Sharing and community curation of mass spectrometry data with Global Natural Products Social Molecular Networking. Nat. Biotechnol..

[48] Sheehan J.C., Zachau H.G., Lawson W.B. (1957). The structure of etamycin. J. Am. Chem. Soc..

[49] Barrow C.J., Oleynek J.J., Marinelli V., Sun H.H., Kaplita P., Sedlock D.M., Gillum A.M., Chadwick C.C., Cooper R. (1997). Antimycins, inhibitors of ATP-citrate lyase, from a *Streptomyces* sp.. J. Antibiot..

[50] Sumner L.W., Amberg A., Barrett D., Beale M.H., Beger R., Daykin C.A., Fan T.W.-M., Fiehn O., Goodacre R., Griffin J.L. (2007). Proposed minimum reporting standards for chemical analysis Chemical Analysis Working Group (CAWG) Metabolomics Standards Initiative (MSI). Metabolomics.

[51] Takada K., Ninomiya A., Naruse M., Sun Y., Miyazaki M., Nogi Y., Okada S., Matsunaga S. (2013). Surugamides A–E, cyclic octapeptides with four d-amino acid residues, from a marine *Streptomyces* sp.: LC–MS-aided inspection of partial hydrolysates for the distinction of D- and L-amino acid residues in the sequence. J. Org. Chem..

[52] Caraballo-Rodríguez A.M., Dorrestein P.C., Pupo M.T. (2017). Molecular inter-kingdom interactions of endophytes isolated from *Lychnophora ericoides*. Sci. Rep..

[53] Matsuda K., Kuranaga T., Sano A., Ninomiya A., Takada K., Wakimoto T. (2019). The revised structure of the cyclic octapeptide surugamide A. Chem. Pharm. Bull..

[54] Gobec S., Frlan R. (2006). Inhibitors of cathepsin B. Curr. Med. Chem..

[55] Velasco-Alzate K.Y., Bauermeister A., Tangerina M.M.P., Lotufo T.M.C., Ferreira M.J.P., Jimenez P.C., Padilla G., Lopes N.P., Costa-Lotufo L.V. (2019). Marine bacteria from Rocas Atoll as a rich source of pharmacologically active compounds. Mar. Drugs.

[56] Neft N., Farley T.M. (1972). Conditions influencing antimycin production by a *Streptomyces* species grown in chemically defined medium. Antimicrob. Agents Chemother..

[57] Abidi S.L. (1982). High-performance liquid chromatographic resolution and quantification of a dilactonic antibiotic mixture (antimycin A). J. Chromatogr..

[58] Guidarelli A., Brambilla L., Rota C., Tomasi A., Cattabeni F., Cantoni O. (1996). The respiratory-chain poison antimycin A promotes the formation of DNA single-strand breaks and reduces toxicity in U937 cells exposed to t-butylhydroperoxide. Biochem. J..

[59] Kim H., Esser L., Hossain M.B., Xia D., Yu C.-A., Rizo J., van der Helm D., Deisenhofer J. (1999). Structure of antimycin A1, a specific electron transfer inhibitor of ubiquinol-cytochrome c oxidoreductase. J. Am. Chem. Soc..

[60] Carcia-Mendoza C. (1965). Studies on the made action of etamycin (viridogrisein). Biochim. Biophys. Acta–Gen. Subj..

[61] Haste N.M., Perera V.R., Maloney K.N., Tran D.N., Jensen P., Fenical W., Nizet V., Hensler M.E. (2010). Activity of the streptogramin antibiotic etamycin against methicillin-resistant *Staphylococcus aureus*. J. Antibiot..

[62] Rothberger J.C. (1901). Ueber die gegenseitigen Beziehungen zwischen curare und physostigmin. Archiv für die gesamte Physiologie des Menschen und der Tiere.

[63] Wright J.L.C. (1984). A new antibiotic from the marine Bryozoan *Flustra foliaceae*. J. Nat. Prod..

[64] Kobayakawa F., Kodani S. (2012). Screening of streptomycetes for production of desferrioxamines. J. Pure Appl. Microbiol..

[65] Patzer S.I., Braun V. (2020). Gene cluster involved in the biosynthesis of griseobactin, a catechol-peptide siderophore of *Streptomyces* sp. ATCC 700974. J. Bacteriol..

[66] Jomon K., Kuroda Y., Ajisaka M., Sakai H. (1972). A new antibiotic, ikarugamycin. J. Antibiot..

[67] Zhang G., Zhang W., Zhang Q., Shi T., Ma L., Zhu Y., Li S., Zhang H., Zhao Y.-L., Shi R. (2014). Mechanistic insights into polycycle formation by reductive cyclization in ikarugamycin biosynthesis. Angew. Chem. Int. Ed. Engl..

[68] Zhou X., Fenical W. (2016). The unique chemistry and biology of the piericidins. J. Antibiot..

[69] Tsakos M., Jacobsen K.M., Yu W., Villadsen N.L., Poulsen T.B. (2016). The rakicidin family of anticancer natural products–synthetic strategies towards a new class of hypoxia-selective cytotoxins. Synlett.

[70] Kitani S., Ueguchi T., Igarashi Y., Leetanasaksakul K., Thamchaipenet A., Nihira T. (2018). Rakicidin F, a new antibacterial cyclic depsipeptide from a marine sponge-derived *Streptomyces* sp.. J. Antibiot..

[71] Kuranaga T., Fukuba A., Ninomiya A., Takada K., Matsunaga S., Wakimoto T. (2018). Diastereoselective total synthesis and structural confirmation of surugamide F. Chem. Pharm. Bull..

[72] Sun P., Maloney K.N., Nam S.-J., Haste N.M., Raju R., Aalbersberg W., Jensen P.R., Nizet V., Hensler M.E., Fenical W. (2011). Fijimycins A–C, three antibacterial etamycin-class depsipeptides from a marine-derived *Streptomyces* sp.. Bioorg. Med. Chem..

[73] Tripathi A., Vázquez-Baeza Y., Gauglitz J.M., Wang M., Dührkop K., Nothias-Esposito M., Acharya D.D., Ernst M., van der Hooft J.J.J., Zhu Q. (2020). Chemically-informed analyses of metabolomics mass spectrometry data with Qemistree. bioRxiv.

[74] Dührkop K., Shen H., Meusel M., Rousu J., Böcker S. (2015). Searching molecular structure databases with tandem mass spectra using CSI:FingerID. Proc. Natl. Acad. Sci. USA.

[75] Caldwell S.L., Laidler J.R., Brewer E.A., Eberly J.O., Sandborgh S.C., Colwell F.S. (2008). Anaerobic Oxidation of Methane: Mechanisms, Bioenergetics, and the Ecology of Associated Microorganisms. Environ. Sci. Technol..

[76] Setianingrum F., Rautemaa-Richardson R., Denning D.W. (2019). Pulmonary cryptococcosis: A review of pathobiology and clinical aspects. Med. Mycol..

[77] Poley M., Koubek R., Walsh L., Mcgillen B. (2019). Cryptococcal meningitis in an apparent immunocompetent patient. J. Investig. Med. High. Impact Case Rep..

[78] Havlickova B., Czaika V.A., Friedrich M. (2008). Epidemiological trends in skin mycoses worldwide. Mycoses.

[79] Roca C., Lehmann M., Torres C.A.V., Baptista S., Gaudêncio S.P., Freitas F., Reis M.A.M. (2016). Exopolysaccharide production by a marine *Pseudoalteromonas* sp. strain isolated from Madeira Archipelago ocean sediments. New Biotechnol..

[80] Mincer T.J., Jensen P.R., Kauffman C.A., Fenical W. (2002). Widespread and persistent populations of a major new marine actinomycete taxon in ocean sediments. Appl. Environ. Microbiol..

[81] Gontang E.A., Fenical W., Jensen P.R. (2007). Phylogenetic diversity of gram-positive bacteria cultured from marine sediments. Appl. Environ. Microbiol..

[82] Waterhouse A.M., Procter J.B., Martin D.M.A., Clamp M., Barton G.J. (2009). Jalview Version 2—A multiple sequence alignment editor and analysis workbench. Bioinformatics.

[83] Katoh K., Misawa K., Kuma K., Miyata T. (2002). MAFFT: A novel method for rapid multiple sequence alignment based on fast Fourier transform. Nucleic Acids Res..

[84] Chun J., Lee J.-H., Jung Y., Kim M., Kim S., Kim B.K., Lim Y.-W. (2007). EzTaxon: A web-based tool for the identification of prokaryotes based on 16S ribosomal RNA gene sequences. Int. J. Syst. Evol. Microbiol..

[85] Zhang Z., Schwartz S., Wagner L., Miller W. (2000). A greedy algorithm for aligning DNA sequences. J. Comput. Biol..

[86] Hall B.G. (2013). Building phylogenetic trees from molecular data with MEGA. Mol. Biol. Evol..

[87] Tamura K., Stecher G., Peterson D., Filipski A., Kumar S. (2013). MEGA6: Molecular Evolutionary Genetics Analysis version 6.0. Mol. Biol. Evol..

[88] Hoff M., Orf S., Riehm B., Darriba D., Stamatakis A. (2016). Does the choice of nucleotide substitution models matter topologically?. BMC Bioinform..

[89] Schloss P.D., Westcott S.L., Ryabin T., Hall J.R., Hartmann M., Hollister E.B., Lesniewski R.A., Oakley B.B., Parks D.H., Robinson C.J. (2009). Introducing mothur: Open-source, platform-independent, community-supported software for describing and comparing microbial communities. Appl. Environ. Microbiol..

[90] Chao A. (1987). Estimating the population size for capture-recapture data with unequal catchability. Biometrics.

[91] Huggins R., Stoklosa J., Roach C., Yip P. (2018). Estimating the size of an open population using sparse capture–recapture data. Biometrics.

[92] Kim B.-R., Shin J., Guevarra R., Lee J.H., Kim D.W., Seol K.-H., Lee J.-H., Kim H.B., Isaacson R. (2017). Deciphering diversity indices for a better understanding of microbial communities. J. Microbiol. Biotechnol..

[93] Aron A., Petras D., Schmid R., Gauglitz J.M., Büttel I., Antelo L., Zhi H., Saak C.C., Malarney K.P., Thines E. (2019). Native electrospray-based metabolomics enables the detection of metal-binding compounds. bioRxiv.

[94] Kessner D., Chambers M., Burke R., Agus D., Mallick P. (2008). ProteoWizard: Open source software for rapid proteomics tools development. Bioinformatics.

[95] Adusumilli R., Mallick P. (2017). Data conversion with ProteoWizard msConvert. Methods Mol. Biol..

[96] Katajamaa M., Miettinen J., Oresic M. (2006). MZmine: Toolbox for processing and visualization of mass spectrometry based molecular profile data. Bioinformatics.

[97] Pluskal T., Castillo S., Villar-Briones A., Orešič M. (2010). MZmine 2: Modular framework for processing, visualizing, and analyzing mass spectrometry-based molecular profile data. BMC Bioinform..

[98] Myers O.D., Sumner S.J., Li S., Barnes S., Du X. (2017). Detailed investigation and comparison of the XCMS and MZmine 2 chromatogram construction and chromatographic peak detection methods for preprocessing mass spectrometry metabolomics data. Anal. Chem..

[99] Shannon P., Markiel A., Ozier O., Baliga N.S., Wang J.T., Ramage D., Amin N., Schwikowski B., Ideker T. (2003). Cytoscape: A software environment for integrated models of biomolecular interaction networks. Genome Res..

[100] Letunic I., Bork P. (2019). Interactive Tree Of Life (iTOL) v4: Recent updates and new developments. Nucleic Acids Res..

