# Supplementary material for: The Diversity, Metabolomics Profiling, and the Pharmacological Potential of Actinomycetes Isolated from the Estremadura Spur Pockmarks (Portugal)
Source: Mar Drugs. 2021 Dec 23;20(1):21. doi: 10.3390/md20010021 (PMC8780274; doi:10.3390/md20010021)
Supplement: Supplementary file 1 [file marinedrugs-20-00021-s001.zip › marinedrugs-1493075-supplementary.pdf]

marine drugs

Supplementary Materials

The Diversity, metabolomics profiling and the pharmacological potential of actinomycetes isolated from the Estremadura Spur pockmarks (Portugal)

António Pinto-Almeida [1,2,3], Anelize Bauermeister [4], Luca Luppino [1,2,5], Inês R. Grilo [1,2], Juliana Oliveira [1,2], Joana R. Sousa [1,2], Daniel Petras [6], Clara F. Rodrigues [7], Alejandra Prieto-Davó [8], Deniz Tasdemir [9,10], Rita G. Sobral [1,2] and Susana P. Gaudêncio [1,2,*]

[1] Associate Laboratory i4HB – Institute for Health and Bioeconomy, NOVA School of Science and Technology, NOVA University Lisbon, 2819-516 Caparica, Portugal
[2] UCIBIO – Applied Molecular Biosciences Unit, NOVA School of Science and Technology, NOVA University of Lisbon, 2819-516 Caparica, Portugal
[3] Instituto de Engenharias e Ciências do Mar, Universidade Técnica do Atlântico, 163 Ribeira de Julião, Mindelo, Cabo Verde
[4] Skaggs School of Pharmacy & Pharmaceutical Science, University of California San Diego, La Jolla, 92093-075 La Jolla, CA,USA
[5] Dipartimento di Scienze Della Vita, Università Degli Studi di Modena e Reggio Emilia, 41125 - Modena, Italia
[6] CMFI Cluster of Excellence, Interfaculty Institute of Microbiology and Medicine, University of Tuebingen, Auf der Morgenstelle 24, 72076 Tuebingen, Germany
[7] CESAM – Centre for Environmental and Marine Studies, Department of Biology, University of Aveiro, 3810-193 Aveiro, Portugal
[8] Unidad de Química-Sisal, Facultad de Química, Universidad Nacional Autónoma de México, Sisal 97356, Mexico
[9] GEOMAR Centre for Marine Biotechnology, Research Unit Marine Natural Products Chemistry, GEOMAR Helmholtz Centre for Ocean Research Kiel, 24106 Kiel, Germany
[10] Faculty of Mathematics and Natural Sciences, Kiel University, Christian-Albrechts-Platz 4, 24118 Kiel, Germany (ORCID ID: 0000-0002-7841-6271)
* Correspondence: e-mail: s.gaudencio@fct.unl.pt (S.P.G.); Tel. (+351) 21 2948300; Fax. (+351) 21 2948550. (ORCID ID: 0000-0002-5510-1170)

Table S1. Estremadura Spur pockmarks stations of sediment collection.

Location	Station_ID	Latitude (N)	Longitude (W)	Depth (m)
Small mound	PES17 L17 D01PC1	39°17′18′′	10°1′3′′	337.8
Small mound	PES17 L17 D01PC3	39°17′21′′	10°1′1′′	339.9
Small mound	PES17 GR01	39°17′23′′	10°1′3′′	345.0
Pockmark	PES17 GR06	39°15′4′′	10°0′57′′	318.0
Pockmark	PES17 GR09	39°15′0′′	9°59′0′′	313.3
Pockmark	PES17 GR14	39°14′41′′	9°59′4′′	289.0
Pockmark	PES17 GR17	39°14′34′	9°59′23′′	279.0
Pockmark	PES17 GR20	39°13′58′′	9°58′55′′	275.0
Pockmark	PES17 GR22	39°12′54′′	9°57′52′′	249.0
Pockmark	PES17 GR23	39°12′49′′	9°56′11′′	237.0
Pockmark	PES17 GR24	39°13′23′′	10°0′20′′	307.0
Pockmark	PES17 GR25	39°13′53′′	10°0′25′′	307.0
Pockmark	PES17 GR26	39°13′23′′	10°0′20′′	307.0
Pockmark	PES17 GR27	39°14′8′′	10°0′22′′	309.0

Table S2. NCBI accession number of the representative 16S rRNA gene sequence of each of the species found as best hit from the search in NCBI rRNA/ITS and EzTaxon databases.

Representative sequences	Species	Strains
NR_041201.1	*Streptomyces gougerotii*	NBRC 3198
NR_108479.1	*Micromonospora zamorensis*	CR38
NR_042312.1	*Micromonospora saelicesensis*	Lupac 09
NR_104522.1	*Micromonospora humi*	P0402
MG725912.1	*Micromonospora* sp.	LB32
NR_042102.1	*Streptomyces griseochromogenes*	ISP 5499
NR_151946.1	*Micromonospora vinacea*	GUI63
NR_116324.1	*Actinopolymorpha cephalotaxi*	I06-2230
NR_041166.1	*Streptomyces aculeolatus*	NBRC 14824
NR_041207.1	*Streptomyces griseolus*	NBRC 3415
NR_041349.1	*Micromonospora chokoriensis*	2-19(6)
NR_152655.1	*Micromonospora profundi*	DS3010
NR_118561.1	*Micromonospora schwarzwaldensis*	HKI0641

NR_036795.1	*Micromonospora chalcea*	1464-217L
NR_153685.1	*Verrucosispora sonchi*	NEAU-QY3
NR_044035.1	*Streptomyces xiamenensis*	MCCC 1A01550
NR_116119.1	*Saccharopolyspora gloriosae*	YIM 60513
NR_044883.1	*Micromonospora echinospora*	DSM 43816
NR_148271.1	*Micromonospora taraxaci*	NEAU-P5
NR_041423.1	*Streptomyces malachitospinus*	NBRC 101004
NR_025015.1	*Micromonospora matsumotoense*	IMSNU 22003
NR_152020.1	*Streptomyces camponoticapitis*	2H-TWYE14
NR_042760.1	*Streptomyces albogriseolus*	DSM 40003
NR_028659.1	*Micromonospora auratinigra*	TT1-11
NR_151945.1	*Micromonospora noduli*	GUI43
NR_041103.1	*Streptomyces intermedius*	NBRC 13049
NR_109059.1	*Actinomadura geliboluensis*	A8036
NR_126175.1	*Streptomyces chumphonensis*	KK1-2
NR_025870.2	*Streptomyces sampsonii*	ATCC 25495
NR_112305.1	*Streptomyces coelicolor*	NBRC 12854
NR_146003.1	*Actinomadura sporangiiformans*	NEAU-Jh2-5
NR_044906.1	*Nocardiopsis prasina*	DSM 43845
NR_152658.1	*Streptomyces ovatisporus*	S4702
NR_116241.1	*Micromonospora tulbaghiae*	TVU1
NR_042059.1	*Saccharomonospora xinjiangensis*	XJ-54 DSM 44391
NR_135859.2	*Stackebrandtia endophytica*	YIM 64602
GU121457.1	*Saccharomonospora* sp.	BCRC 16893
NR 074415.1	*Micromonospora aurantiaca*	ATCC 27029
NR 044886.1	*Micromonospora citrea*	DSM 43903
NR_044148.1	*Streptomyces halstedii*	NRRL B-1238
NR 114960.1	*Streptomyces marokkonensis*	LMG 23016
NR 041095.1	*Streptomyces albidoflavus*	NBRC 13010
MN255488.1	*Streptomyces hydrogenans*	YAM1
AB184868.1	*Streptomyces hydrogenans*	NBRC 13475

Table S3. List of all strains isolated from the Estremadura Spur, continental Portugal coast, including their station, habitat, depth, seawater requirement and accession number from GenBank (AccessID).

PTE	Station	Habitat	Depth (m)	Seawater requirement	AccessID
PTE-001	PESGR01	Small mound	345	No	MT830750
PTE-002	PESGR14	Pockmark	289	No	MT830751
PTE-003	PESGR17	Pockmark	279	No	MT830752
PTE-004	PESGR17	Pockmark	279	No	MT830753
PTE-005	PESGR22	Pockmark	249	No	MT830754
PTE-006	PESGR25	Pockmark	307	No	MT830755
PTE-007	PESGR24	Pockmark	307	No	MT830756
PTE-008	PESGR17	Pockmark	279	No	MT830757
PTE-009	PESGR20	Pockmark	275	No	MT830758
PTE-010	PESGR01	Small mound	345	No	MT830759
PTE-011	PESGR06	Pockmark	318	No	MT830760
PTE-012	PESGR01	Small mound	345	No	MT830761
PTE-013	PESGR17	Pockmark	279	No	MT830762
PTE-014	PESGR20	Pockmark	275	No	MT830763
PTE-015	PESGR27	Pockmark	309	No	MT830764
PTE-016	PESGR25	Pockmark	307	No	MT830765
PTE-017	PESGR20	Pockmark	275	No	MT830766
PTE-018	PESGR23	Pockmark	237	No	MT830767
PTE-019	PESGR23	Pockmark	237	No	MT830768
PTE-020	PESGR17	Pockmark	279	No	MT830769
PTE-021	PESGR17	Pockmark	279	No	MT830770
PTE-022	D01PC1	Small mound	337.8	No	MT830771
PTE-023	PESGR25	Pockmark	307	No	MT830772
PTE-024	PESGR27	Pockmark	309	No	MT830773
PTE-025	PESGR20	Pockmark	275	No	MT830774
PTE-026	PESGR20	Pockmark	275	No	MT830775
PTE-027	PESGR22	Pockmark	249	No	MT830776
PTE-028	PESGR01	Small mound	345	No	MT830777
PTE-029	PESGR22	Pockmark	249	No	MT830778
PTE-030	PESGR26	Pockmark	307	No	MT830779
PTE-031	PESGR20	Pockmark	275	No	MT830780
PTE-032	PESGR20	Pockmark	275	No	MT830781
PTE-033	PESGR22	Pockmark	249	No	MT830782

PTE-034	PESGR17	Pockmark	279	No	MT830783
PTE-035	PESGR17	Pockmark	279	No	MT830784
PTE-036	PESGR20	Pockmark	275	No	MT830785
PTE-037	PESGR23	Pockmark	237	No	MT830786
PTE-038	PESGR23	Pockmark	237	yes	MT830787
PTE-039	PESGR17	Pockmark	279	No	MT830788
PTE-040	PESGR17	Pockmark	279	No	MT830789
PTE-041	PESGR01	Small mound	345	No	MT830790
PTE-042	PESGR20	Pockmark	275	No	MT830791
PTE-043	PESGR06	Pockmark	318	No	MT830792
PTE-044	PESGR25	Pockmark	307	No	MT830793
PTE-045	PESGR25	Pockmark	307	No	MT830794
PTE-046	PESGR25	Pockmark	307	yes	MT830795
PTE-047	PESGR01	Small mound	345	No	MT830796
PTE-048	PESGR01	Small mound	345	yes	MT830797
PTE-049	PESGR20	Pockmark	275	No	MT830798
PTE-050	PESGR20	Pockmark	275	No	MT830799
PTE-051	PESGR17	Pockmark	279	No	MT830800
PTE-052	PESGR06	Pockmark	318	No	MT830801
PTE-053	PESGR20	Pockmark	275	No	MT830802
PTE-054	PESGR22	Pockmark	249	yes	MT830803
PTE-055	PESGR25	Pockmark	307	No	MT830804
PTE-056	PESGR25	Pockmark	307	yes	MT830805
PTE-057	PESGR17	Pockmark	279	yes	MT830806
PTE-058	PESGR17	Pockmark	279	yes	MT830807
PTE-059	PESGR23	Pockmark	237	yes	MT830808
PTE-060	PESGR06	Pockmark	318	No	MT830809
PTE-061	PESGR17	Pockmark	279	yes	MT830810
PTE-062	D01PC1	Small mound	337.8	yes	MT830811
PTE-063	PESGR23	Pockmark	237	yes	MT830812
PTE-064	PESGR17	Pockmark	279	No	MT830813
PTE-065	PESGR23	Pockmark	237	yes	MT830814
PTE-066	PESGR09	Pockmark	313.3	yes	MT830815
PTE-067	D01PC1	Small mound	337.8	No	MT830816
PTE-068	PESGR22	Pockmark	249	No	MT830817
PTE-069	D01PC1	Small mound	337.8	No	MT830818

PTE-070	PESGR23	Pockmark	237	yes	MT830819
PTE-071	PESGR20	Pockmark	275	No	MT830820
PTE-072	PESGR23	Pockmark	237	yes	MT830821
PTE-073	PESGR23	Pockmark	237	yes	MT830822
PTE-074	PESGR23	Pockmark	237	No	MT830823
PTE-075	D01PC1	Small mound	337.8	No	MT830824
PTE-076	PESGR23	Pockmark	237	No	MT830825
PTE-077	D01PC3	Small mound	339.9	No	MT830826
PTE-078	PESGR23	Pockmark	237	No	MT830827
PTE-079	PESGR20	Pockmark	275	No	MT830828
PTE-080	PESGR20	Pockmark	275	No	MT830829
PTE-081	PESGR23	Pockmark	237	yes	MT830830
PTE-082	PESGR26	Pockmark	307	No	MT830831
PTE-083	PESGR17	Pockmark	279	No	MT830832
PTE-085	PESGR23	Pockmark	237	yes	MT830833
PTE-086	PESGR23	Pockmark	237	No	MT830834

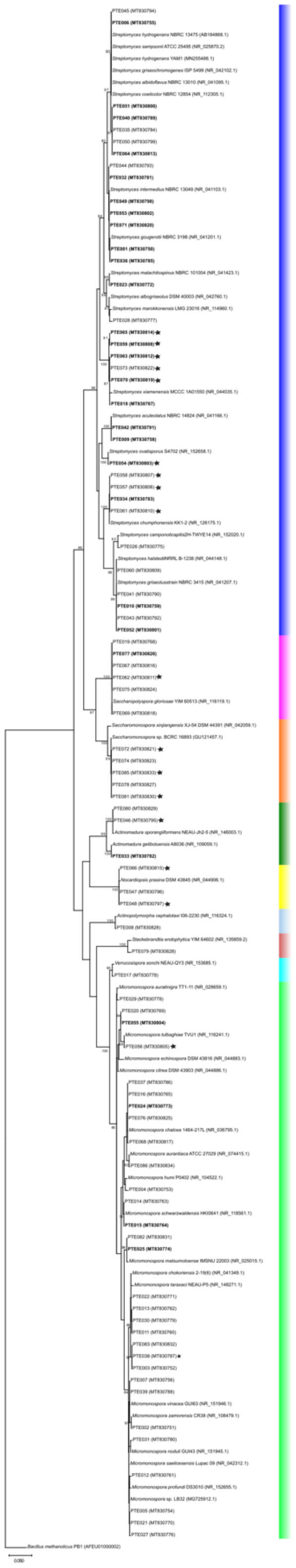

Figure S1. Maximum Likelihood phylogenetic tree of the alignment of the 16S rRNA gene from all (85) actinomycetes isolates from Estremadura Spur, continental Portugal, and the representative sequences of all species retrieved from the GenBank (Table S2). The tree was created using 1000 bootstraps. Nodes above 80%

bootstrap values are shown. GenBank accession numbers are indicated after the sequence name. All PTE numbers refer to internal reference collection codes. Stars represent the strains with seawater requirement for growth. Bioactive PTE strains are shown in bold. *Bacillus methanolicus* was used as outgroup.

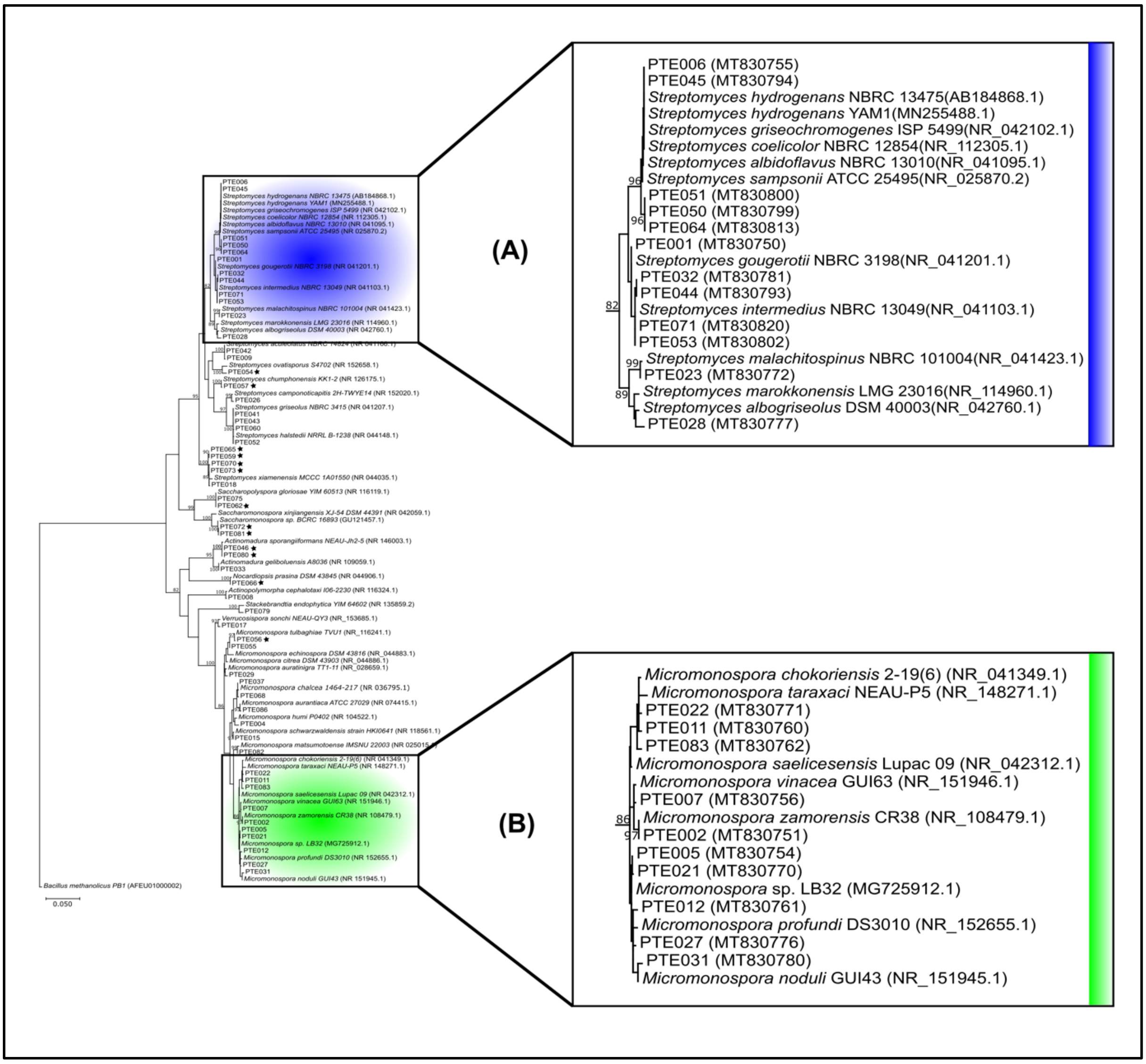

Figure S2. Maximum Likelihood phylogenetic tree of the alignment of the 16S rRNA gene from *Streptomyces* (A) and *Micromonospora* (B) isolates from Estremadura Spur, continental Portugal, and the representative sequences of all species of the respective genus retrieved from the GenBank (Table S2). Regions where it was not possible to distinguish the different lineages based on this genetic marker are highlighted. This tree was generated using 1000 bootstraps.

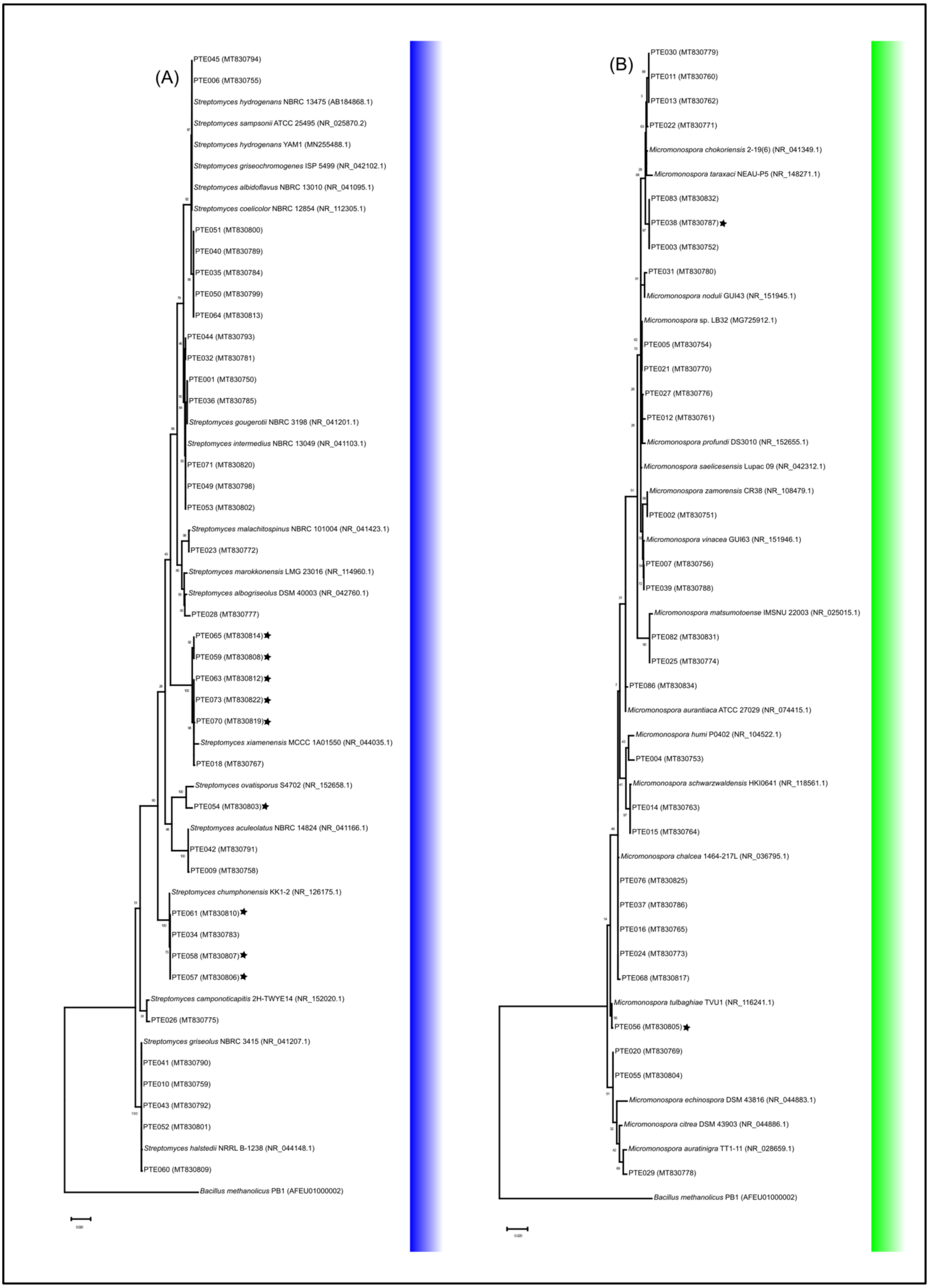

Figure S3. Maximum Likelihood phylogenetic trees of the alignments of the 16S rRNA gene from *Streptomyces* (A) and *Micromonospora* (B) isolates from Estremadura Spur, continental Portugal, and the representative sequences of all species of the respective genus retrieved from the GenBank (Table S2). This tree shows that

removing other genera from the alignment does not allow a better clustering when compared to **Figure S2**. excluding the effect of long-branch attraction. This tree was generated using 1000 bootstraps. Stars represent the strains with seawater requirement for growth.

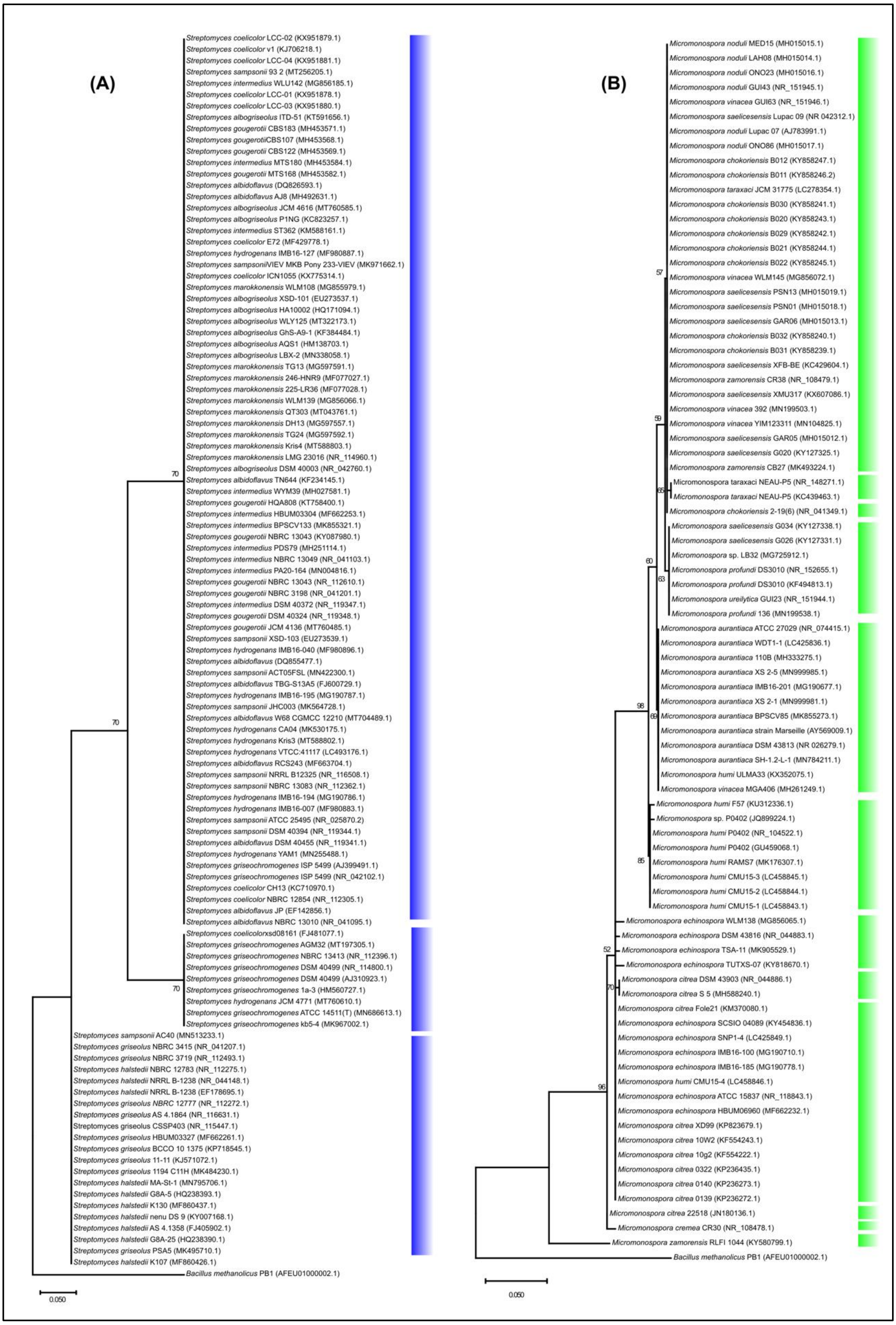

Figure S4. Maximum Likelihood phylogenetic trees of the alignments of the 16S rRNA gene from *Streptomyces* (A) and *Micromonospora* (B) isolates retrieved from GenBank. This tree shows that the multiple isolates of different species of the same genus cluster together. This result discards the possibility that misidentifications

influenced Figure S3 and reflects a low ability to distinguish the species of these genera based on this genetic marker. The tree was created using 1000 bootstraps. Stars represent the strains with seawater requirement for growth.

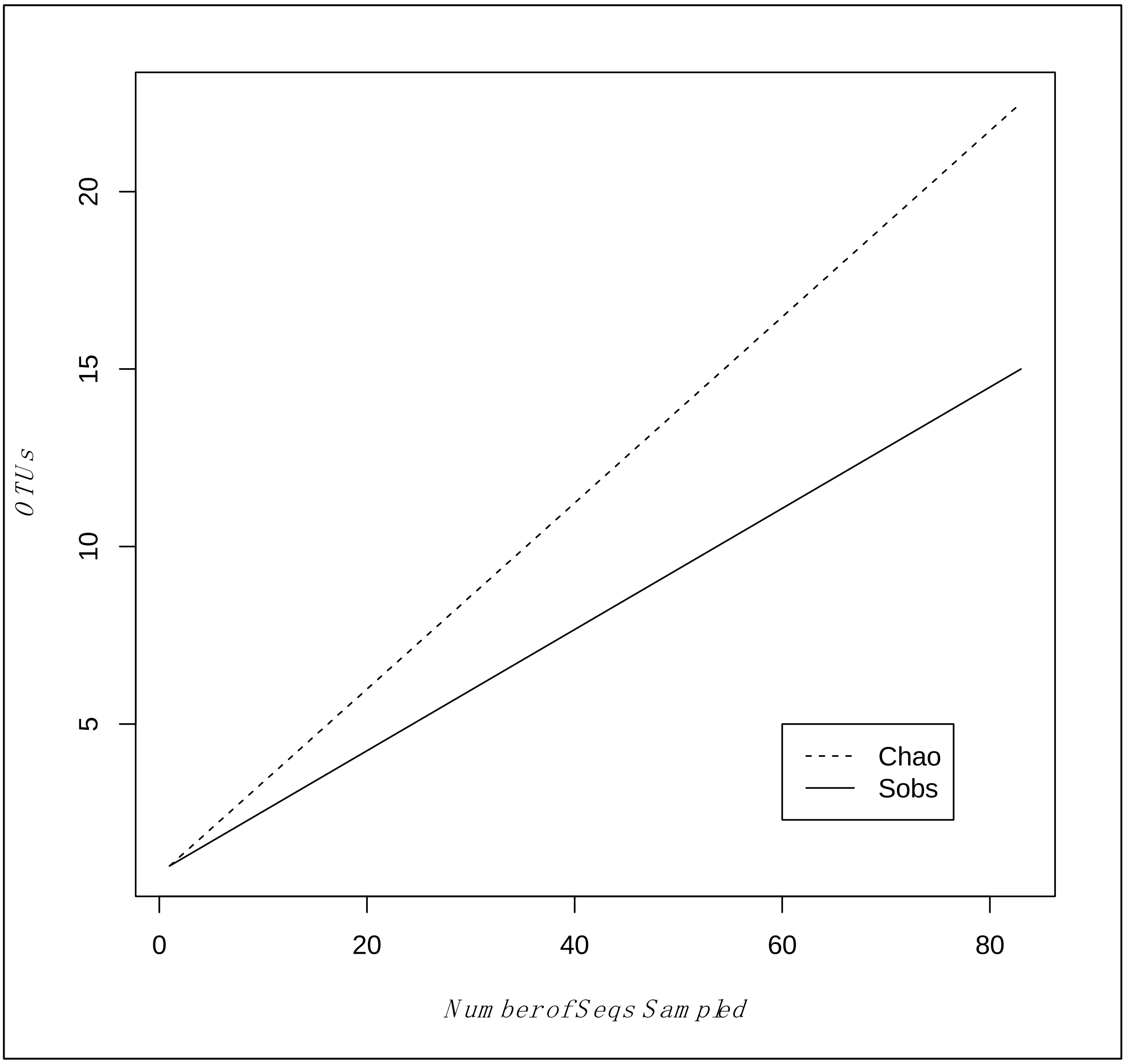

Figure S5. Rarefaction curves for richness (sobs) and estimated richness (chao) using the statistical estimator Chao 1 [43] of isolated marine derived actinomycetes from the Estremadura Spur, continental Portugal. A plateau of the data was not reached, which suggests that further sampling or processing of samples is needed to achieve a higher coverage of the cultivable actinomycete richness from the Estremadura Spur, Continental Portugal.

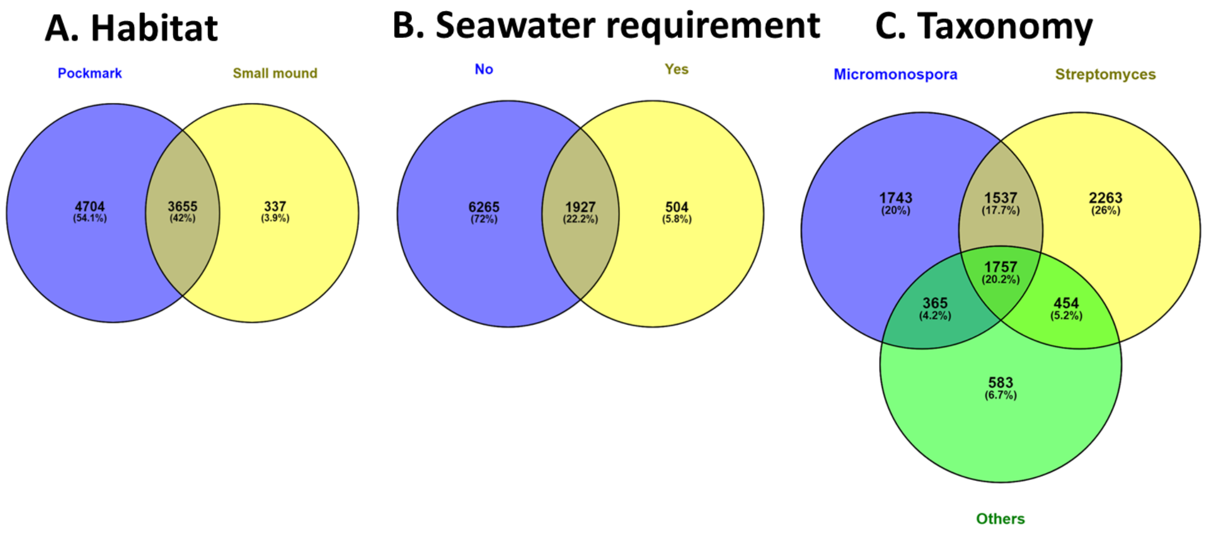

Figure S6: Venn diagram constructed with the ions detected in the crude extracts. The colours are according to legend.

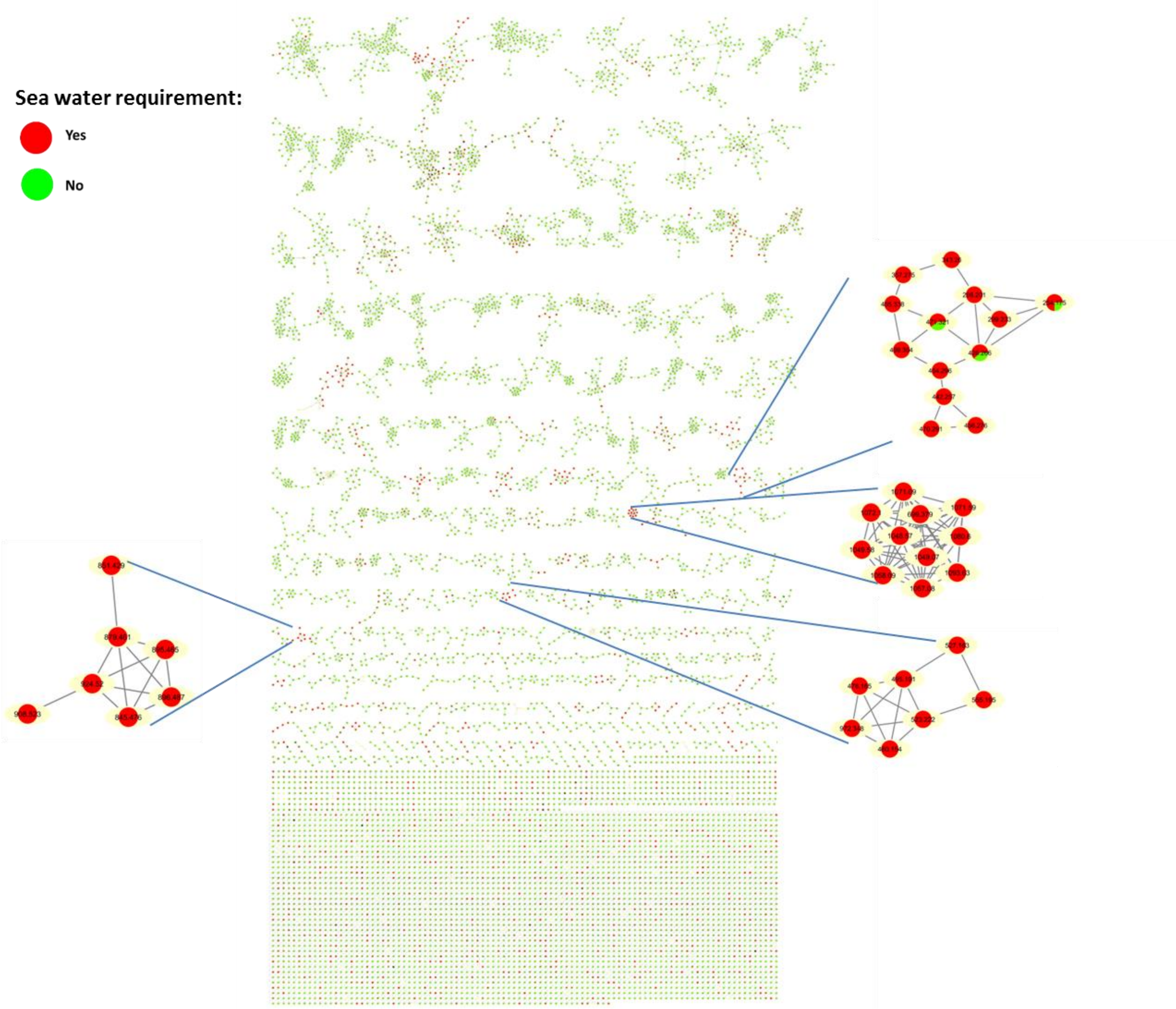

Figure S7. Molecular Networking for Estremadura Spur actiomycetes strains that require sea water for their growth. MS/MS data with positive ionization mode (ESI+). Node colours represent the sea water requirement for strain growth according to the legend. Nodes represent parent ions, and edge strength indicates the chemical similarity between the MS/MS spectra. The node size indicates the number of MS/MS spectra. Only clusters containing at least two nodes are shown. Clusters of metabolites produced exclusively from sea water requiring strains highlighted in red.
